# Supplementary material for: Regulatory pathways governing murine coronary vessel formation are dysregulated in the injured adult heart
Source: Nat Commun. 2019 Jul 22;10:3276. doi: 10.1038/s41467-019-10710-2 (PMC6646353; doi:10.1038/s41467-019-10710-2)
Supplement: Supplementary file 1 — Supplementary Information [file 41467_2019_10710_MOESM1_ESM.pdf]

Supplementary Information

Regulatory pathways governing murine coronary vessel formation are dysregulated in the injured adult heart

Payne et al.

Supplementary Figure 1

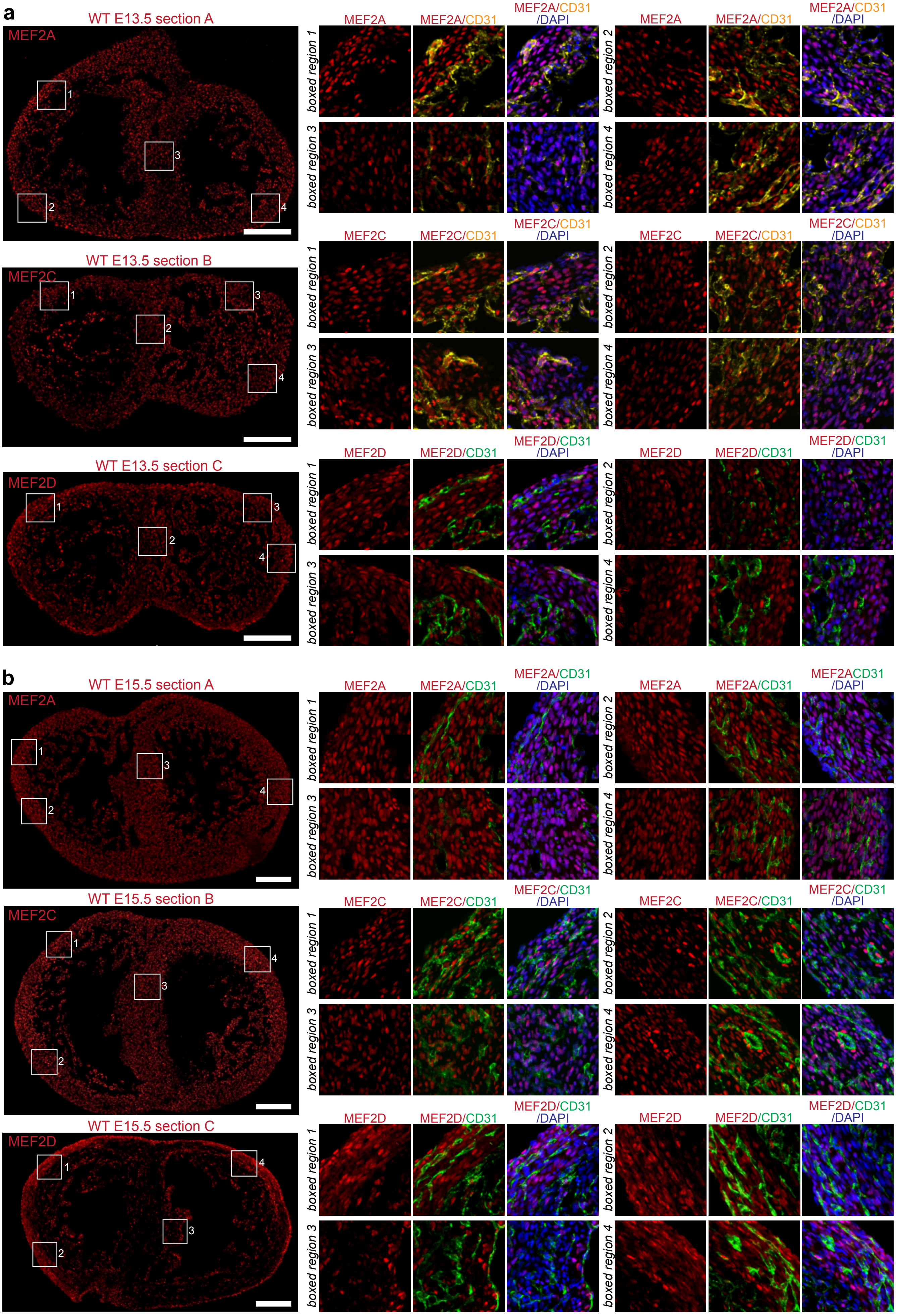

Supplementary Figure 1, relating to Figures 1 and 2 in main text.

Expression of the MEF2A MEF2C and MEF2D transcription factors in the heart at E13.5 and E15.5.

Representative images of immunostaining for MEF2A, MEF2C or MEF2D was compared to the pan endothelial marker CD31 and nuclear DAPI expression on transverse heart sections at E13.5 (**a**) and E15.5 (**b**). At E13.5, all three transcription factors showed widespread expression and nuclear localisation throughout the embryonic heart and in the coronary vasculature. All three factors were also expressed in some CD31-positive endothelial cells at E15.5, although MEF2C was more consistently found in endothelial cell nuclei than MEF2A and MEF2D. White scale bars represent 200µm. MEF2A image representative of two (E13.5) and three (E15.5) biologically independent experiments, MEF2C image representative of five (E13.5) and six (E15.5) biologically independent experiments, MEF2D image representative of three (E13.5) and two (E15.5) biologically independent experiments.

Supplementary Figure 2

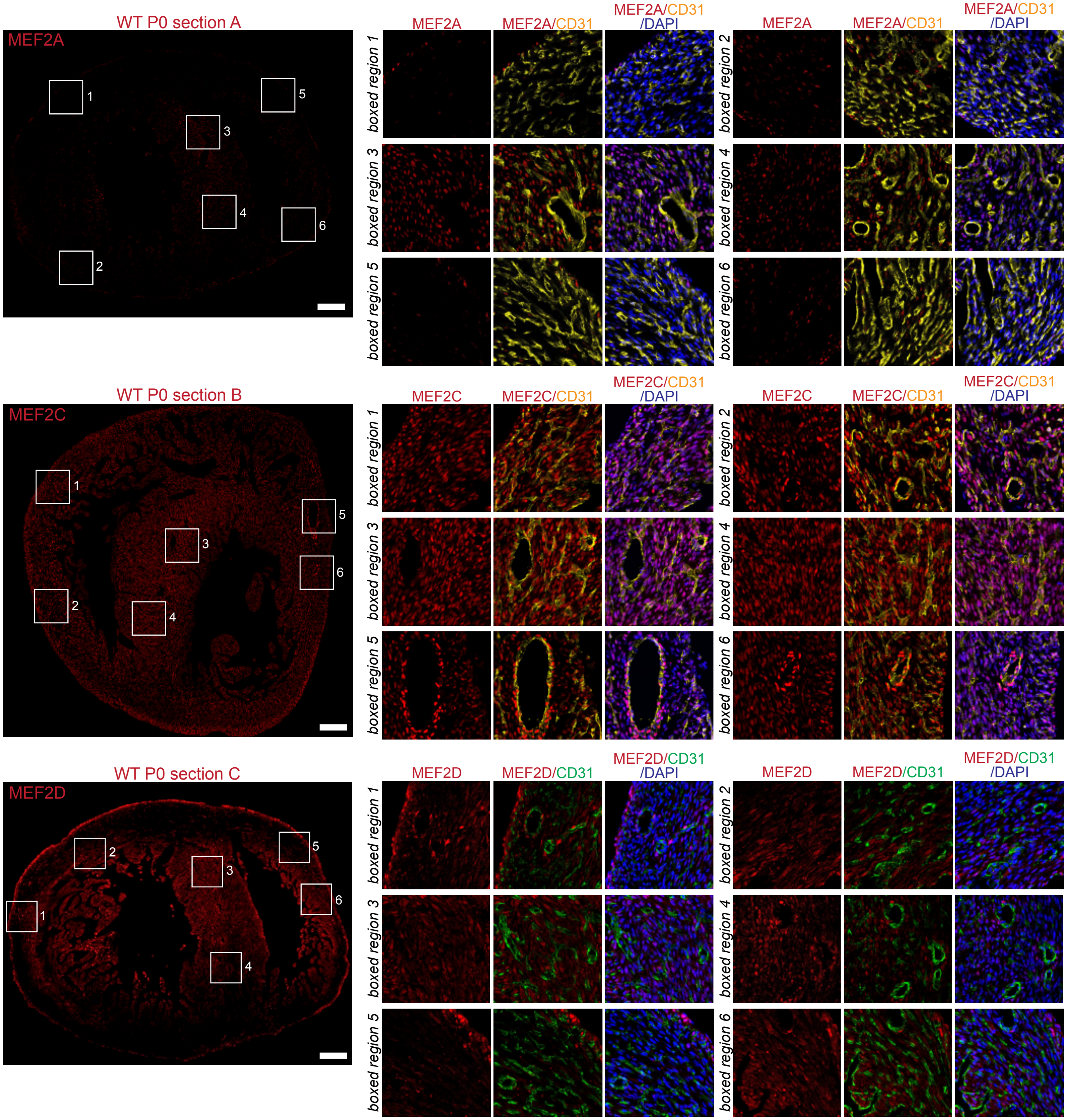

Supplementary Figure 2 relating to Figures 1 and 2 in main text

Expression of the MEF2A MEF2C and MEF2D transcription factors in the P0 heart
Representative images of immunostaining for MEF2A, MEF2C or MEF2D was compared to the pan-endothelial marker CD31 and nuclear DAPI expression on transverse heart sections at P0. All three transcription factors show continued nuclear expression in the neonatal heart, with MEF2C showing the most expression within endothelial cell nuclei. All images represent at least three biologically independent experiments.

Supplementary Figure 3

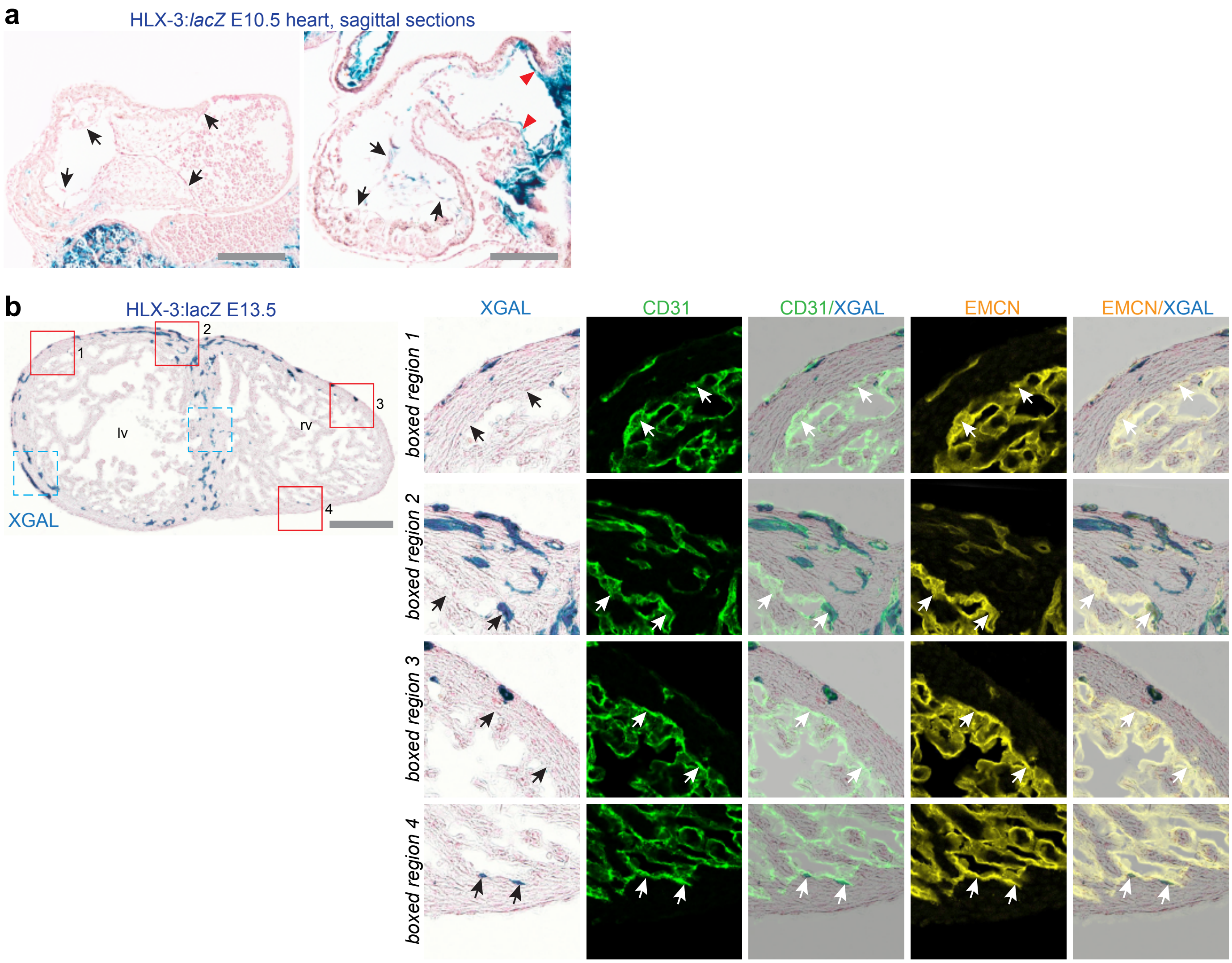

Supplementary Figure 3 relating to Figures 1 and 2 in main text

Analysis of HLX-3:*lacZ* enhancer activity in the E13.5 heart
(**a**) Representative sagittal sections through the E10.5 HLX-3:*lacZ* heart (from three biologically independent embryos, no variation seen). Transgene activity is seen in some endocardium (black arrows) and in the lining of the outflow tract (red arrowheads).
(**b**) Further views of the transverse section through a E13.5 HLX-3:*lacZ* heart shown in Fig. 1d (representative of four similar biologically independent experiments). Enhancer activity, represented by blue X-gal staining, is compared with immunostaining for markers of all endothelial cells (CD31) and mature vein/endocardium (EMCN). Most CD31-positive vessels show HLX-3 enhancer activity, whilst a subset of endocardial cells also show enhancer activity. Blue boxes indicate regions shown in Fig. 1d.
Grey scale bars represent 200μm. Black/white arrows indicate the endocardium. rv, right ventricle; lv, left ventricle.

Supplementary Figure 4

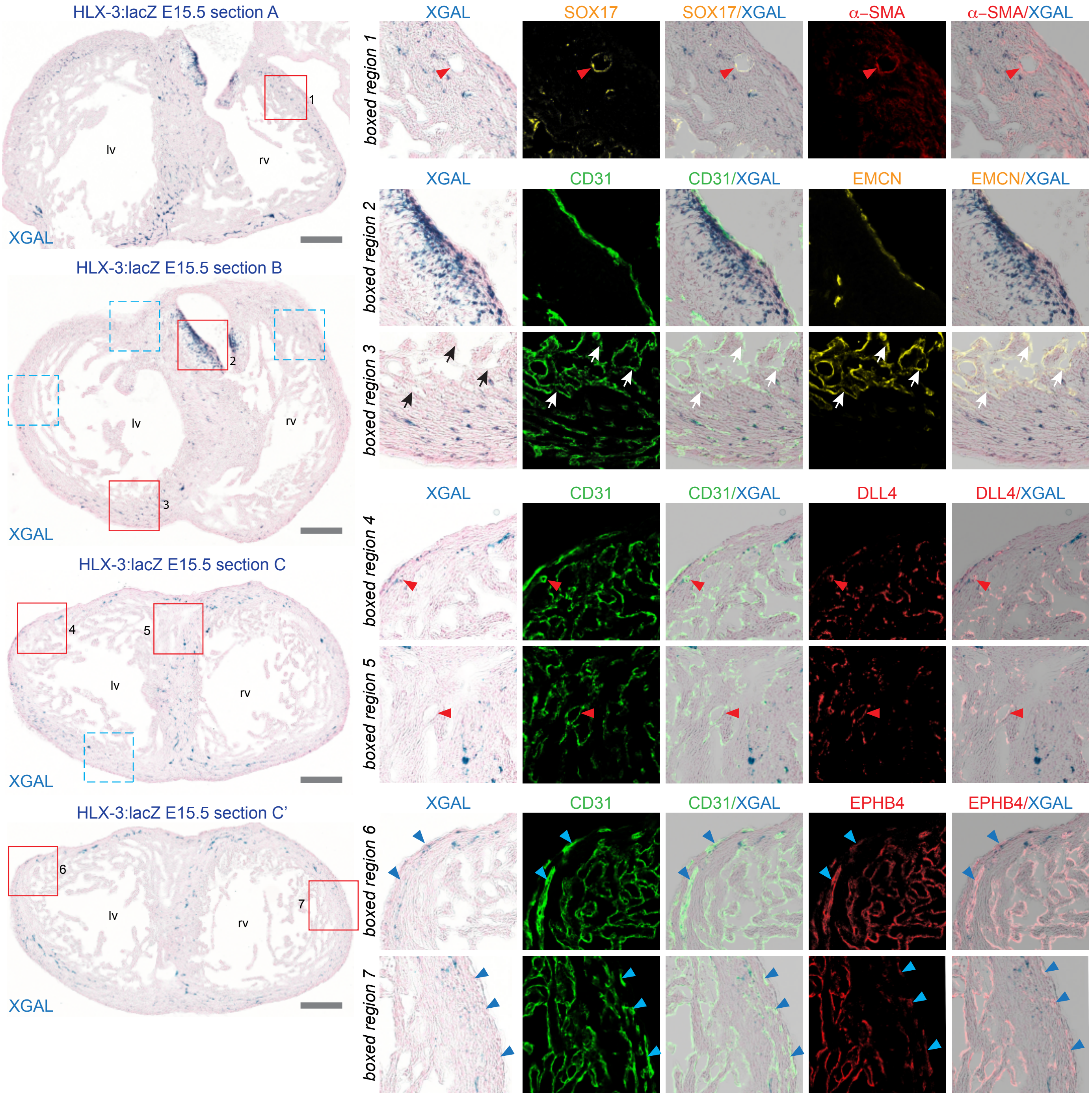

Supplementary Figure 4 relating to Figures 1 and 2 in main text

Analysis of HLX-3:*lacZ* enhancer activity in the E15.5 heart
Further views of transverse sections through the E15.5 HLX-3:*lacZ* heart shown in Fig. 1e (representative of seven similar biologically independent experiments), comparing enhancer activity represented by blue X-gal staining with immunostaining for markers of all endothelial cells (CD31), arteries (SOX17 and α-SMA), angiogenic cells and endocardium (DLL4), and mature veins/endocardium (EMCN and EPHB4) . Blue boxes indicate regions shown in Fig. 1e, grey scale bars represent 200μm. Black/white arrows indicate the endocardium, red arrowheads indicate arteries and blue arrowheads indicate veins. rv, right ventricle; lv, left ventricle.

Supplementary Figure 5

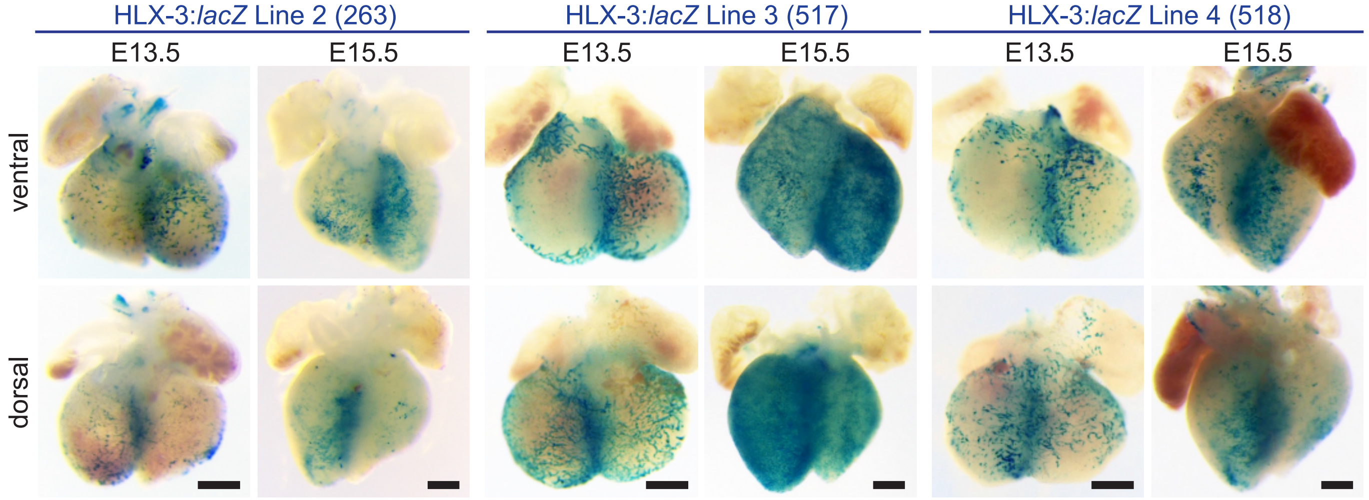

Supplementary Figure 5 relating to Figures 1 and 2 in main text

Independent HLX-3:*lacZ* transgenic lines show consistent patterns of enhancer activity
Representative (of at least four biologically independent samples) E13.5 and E15.5 whole-mount images of hearts from three additional stable transgenic mouse lines independently expressing the HLX-3:*lacZ* transgene, demonstrating similar patterns of enhancer activity at each time-point. Black scale bars represent 500μm.

Supplementary Figure 6

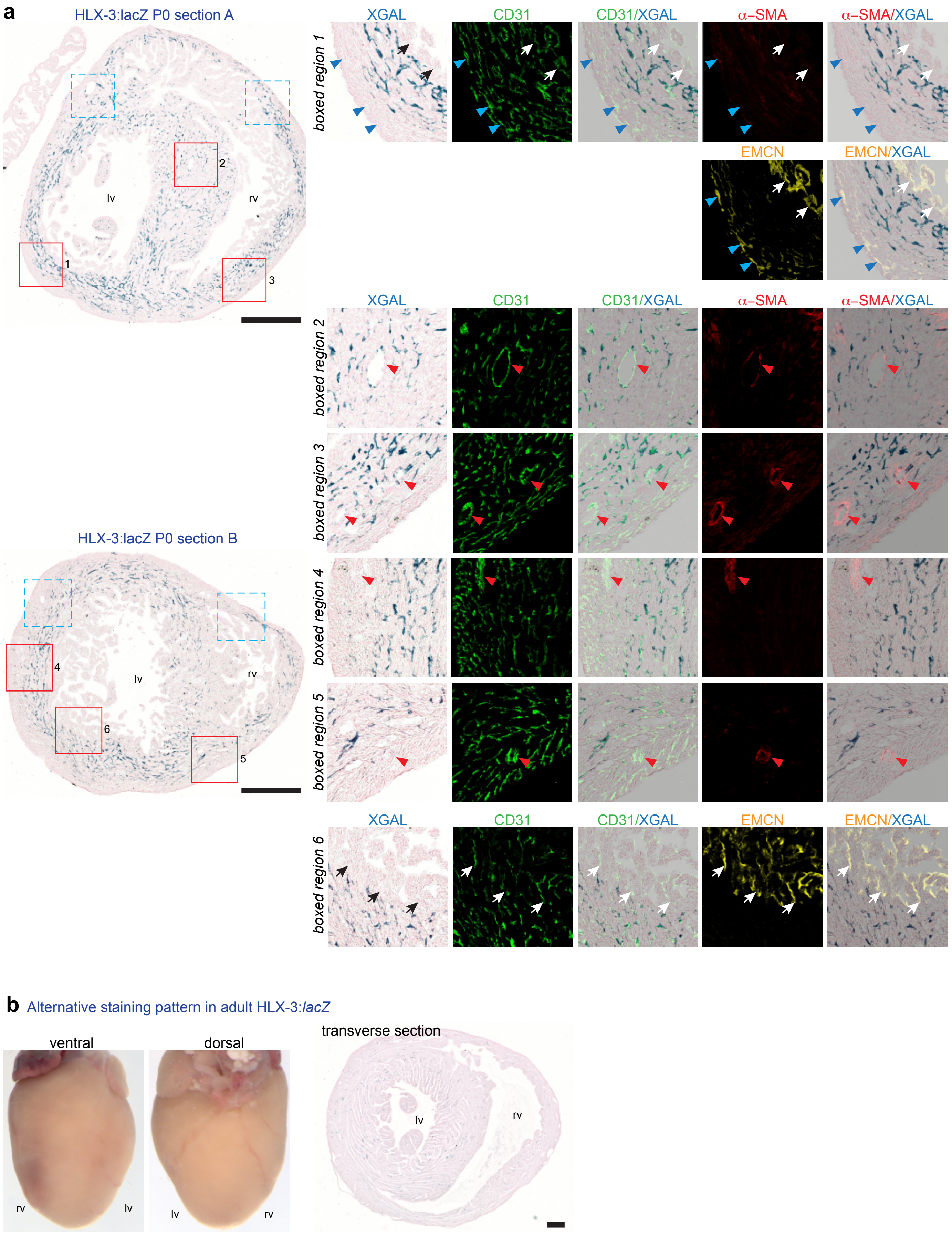

Supplementary Figure 6, relating to Figures 1 and 2 in main text.

Further analysis of HLX-3:*lacZ* enhancer activity in the P0 heart

(**a**) Further views of the transverse sections through a P0 HLX-3:*lacZ* heart shown in Fig. 2b (representative of three similar biologically independent experiments) comparing HLX-3 enhancer activity represented by blue X-gal staining with immunostaining for markers of all endothelial cells (CD31), arteries (α-SMA), and veins (EMCN). Blue boxes indicate regions shown in Fig. 2b.

(**b**) Some adult HLX-3:lacZ hearts showed less X-gal staining than the image shown in Fig. 2d, although the pattern of transgene expression was similar. This is a representative image of this alternative intensity of X-gal staining.

Scale bars represent 500μm. Black/white arrows indicate the endocardium, red arrowheads indicate arteries and blue arrowheads indicate veins. rv, right ventricle; lv, left ventricle.

Supplementary Figure 7

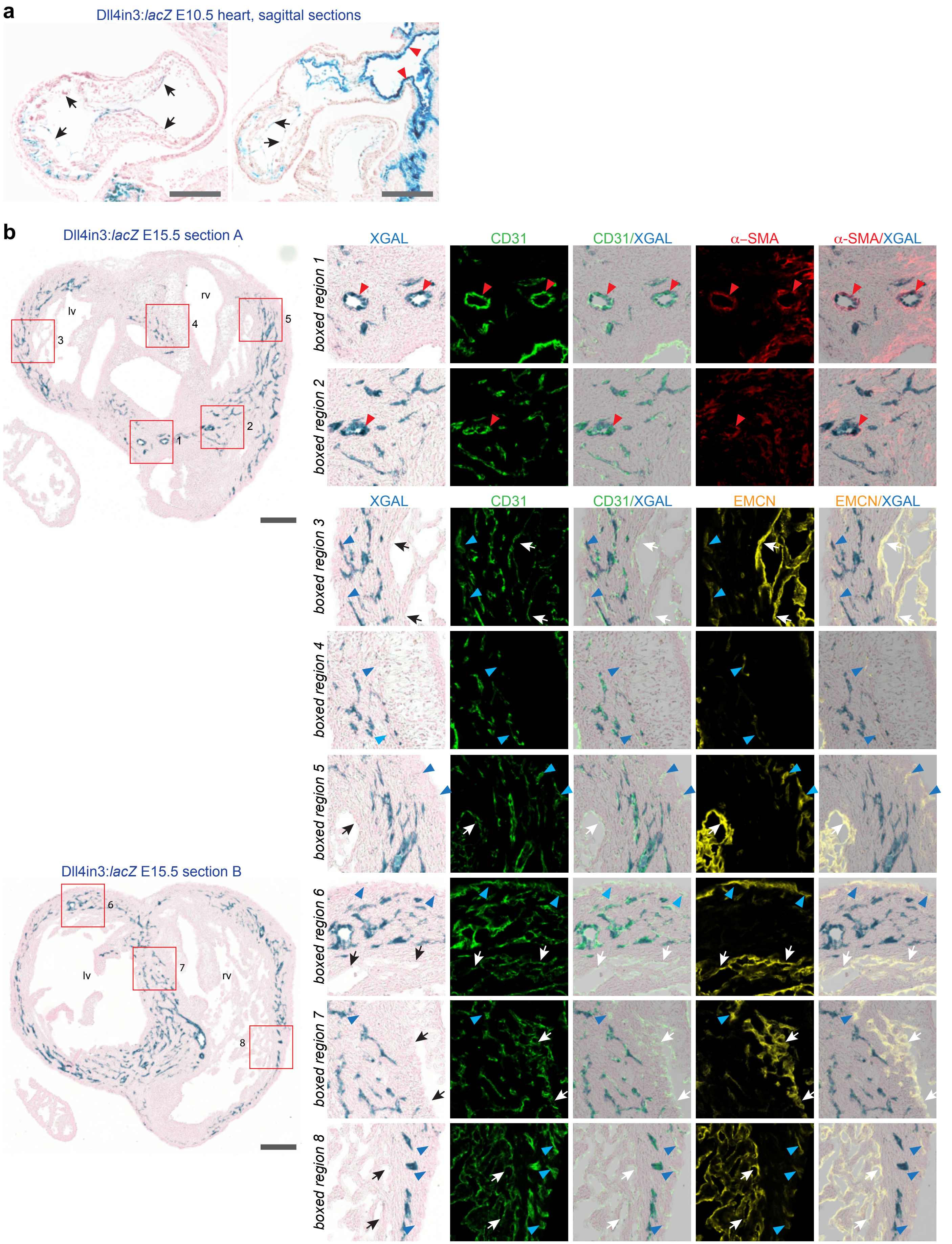

Supplementary Figure 7 relating to Figure 3 in main text

Analysis of Dll4in3:*lacZ* enhancer activity in the E15.5 heart

(**a**) Representative sagittal sections through the E10.5 Dll4in3:*lacZ* heart (from three independent samples, no variation seen). Robust expression is seen in the outflow tract (red arrowheads) and weaker expression seen in parts of the endocardium (black arrows).

(**b**) Sections through an E15.5 Dll4in3:*lacZ* transgenic heart (representative of six similar biologically independent experiments), with blue X-gal staining showing enhancer activity comparative to immunostaining for markers of all endothelial cells (CD31), arteries (α-SMA) and veins/endocardium (EMCN). The Dll4in3 enhancer is active in α-SMA-positive arteries, and some smaller CD31-positive vessels. No activity was seen in the endocardium or venous endothelium.

Black/white arrows indicate the endocardium, red arrowheads indicate arteries, blue arrowheads indicate veins. rv, right ventricle; lv, left ventricle. Grey scale bars represent 200μm.

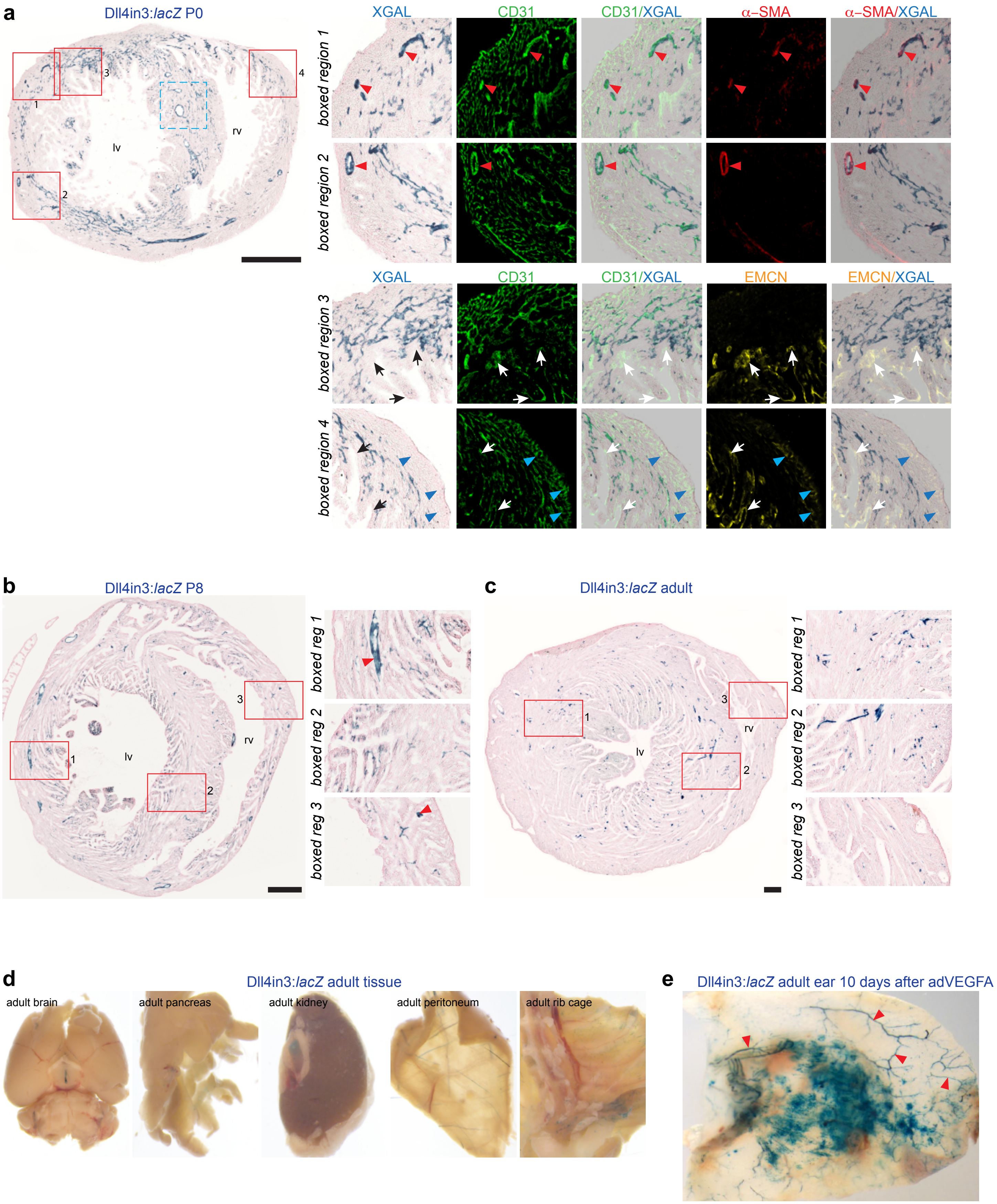
a
Dll4in3:lacZ P0
1
2
3
4
lv
rv
XGAL
CD31
CD31/XGAL
α–SMA
α–SMA/XGAL
boxed region 1
boxed region 2
XGAL
CD31
CD31/XGAL
EMCN
EMCN/XGAL
boxed region 3
boxed region 4
b
Dll4in3:lacZ P8
lv
rv
boxed reg 1
boxed reg 2
boxed reg 3
c
Dll4in3:lacZ adult
lv
rv
boxed reg 1
boxed reg 2
boxed reg 3
d
Dll4in3:lacZ adult tissue
adult brain
adult pancreas
adult kidney
adult peritoneum
adult rib cage
e
Dll4in3:lacZ adult ear 10 days after adVEGFA

Supplementary Figure 8 relating to Figure 3 in main text

Further analysis of Dll4in3:*lacZ* enhancer activity in the postnatal heart
(**a**) Further magnified regions from the transverse section through a P0 Dll4in3:*lacZ* transgenic heart, as shown in Fig. 3c (representative of three similar biologically independent experiments) Enhancer activity visualized by X-gal staining is compared to markers for all endothelial cells (CD31), arteries (α-SMA) and veins/endocardium (EMCN). Blue boxes indicate regions shown in Fig. 3c.
(**b-c**) Transverse sections through a P8 (b) and adult (c) Dll4i3:*lacZ* transgenic heart (representative of at least 5 similar biologically independent samples), demonstrating Dll4in3 activity is maintained into adulthood. At P8 transgene activity is seen in large arteries and smaller vessels, but in the adult transgene activity is mainly restricted to small vessels, predominantly in the inner myocardium, similar to that seen with HLX-3:*lacZ*.
(**d-e**) Dll4in3:*lacZ* activity in adult tissue. Little transgene activity is detected in mature arteries in adult organs (d), but Dll4in3:*lacZ* can be reactivated by adVEGFA^{165} injection in adult ear (e).
Black/white arrows indicate the endocardium, red arrowheads indicate arteries. rv, right ventricle; lv, left ventricle. Black scale bars represent 500μm.

Supplementary Figure 9

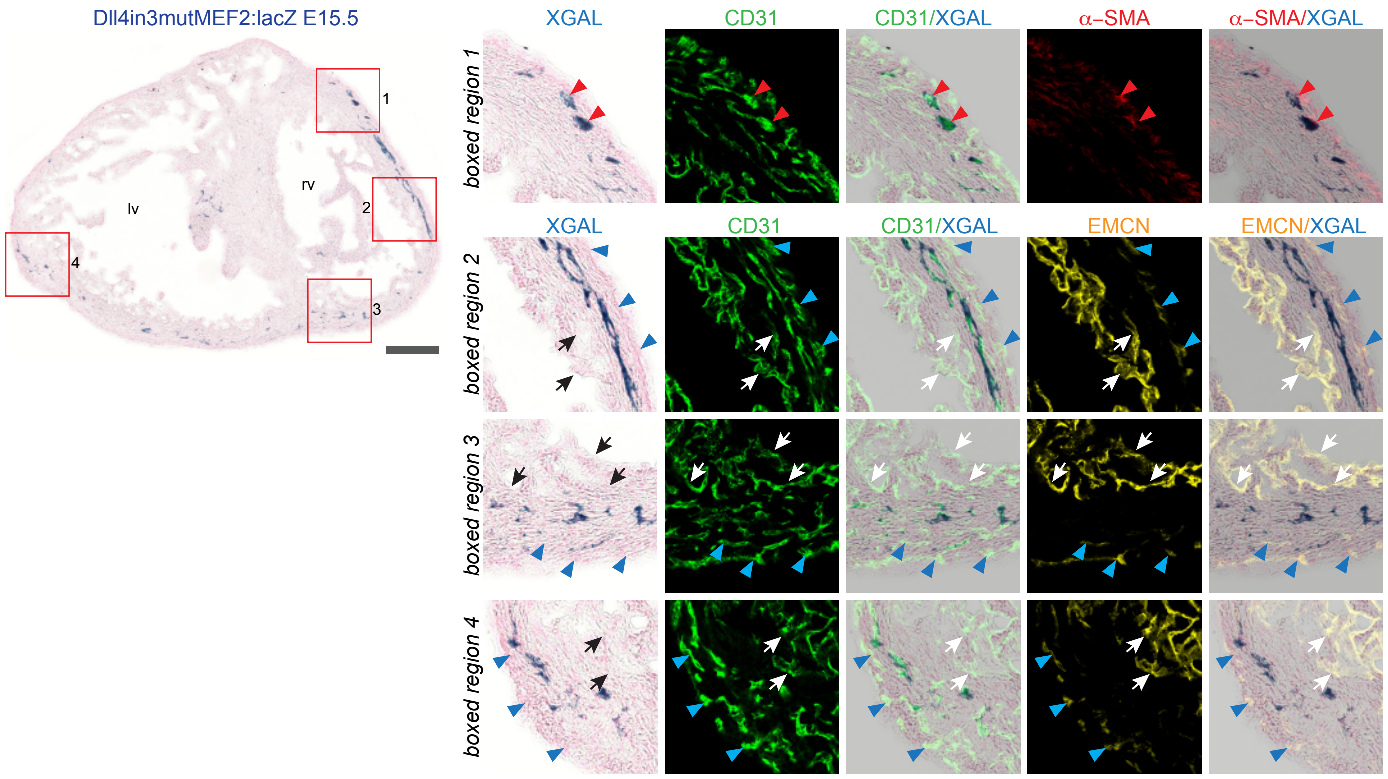

Supplementary Figure 9 relating to Figure 3 in main text.

Analysis of Dll4in3mutMEF:*lacZ* enhancer activity in the E15.5 heart
Transverse section through an E15.5 Dll4in3mutMEF:*lacZ* transgenic heart (representative of nine biologically independent samples), with blue X-gal staining showing enhancer activity compared to immunostaining for markers of all endothelial cells (CD31), arteries (α-SMA) and veins/endocardium (EMCN). Like Dll4in3, the Dll4i3mutMEF enhancer is active in α-SMA-positive arteries, and absent in EMCN-positive endocardium and veins. However, most of the additional activity in smaller CD31-positive vessels throughout the septum and ventricular walls of Dll4in3:*lacZ* transgenic hearts is absent in the Dll4i3mutMEF:*lacZ* heart. Black/white arrows indicate the endocardium, red arrowheads indicate arteries, blue arrowheads indicate veins. rv, right ventricle; lv, left ventricle. Grey scale bars represent 200μm.

Supplementary Figure 10

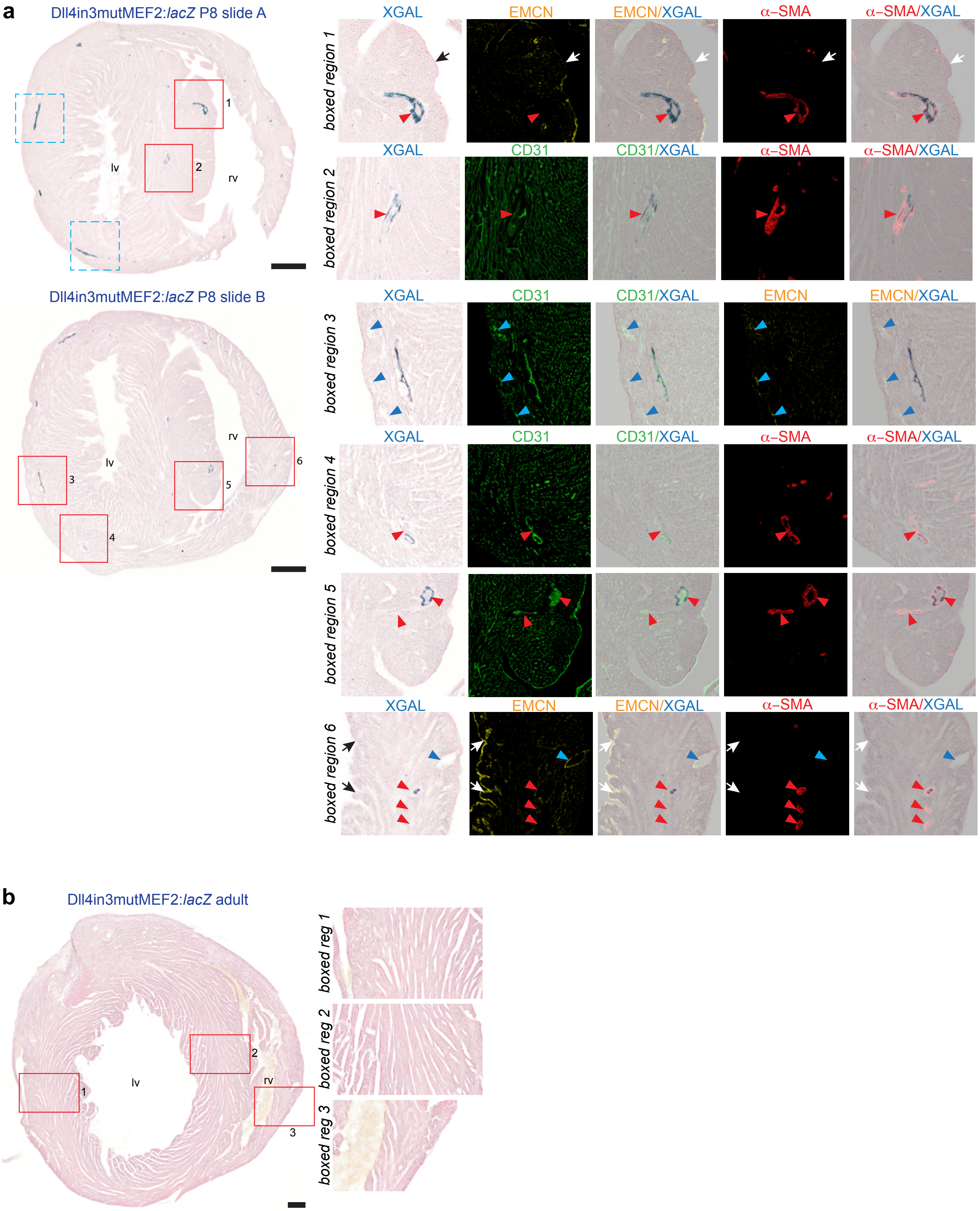

Supplementary Figure 10 relating to Figure 3 in main text

Further analysis of Dll4in3mutMEF:*lacZ* enhancer activity in the postnatal heart
(**a**) Further magnified regions from the transverse section through a P8 Dll4in3mutMEF2:*lacZ* transgenic heart, as shown in Fig. 3f (representative of four similar biologically independent experiments). Enhancer activity is compared to markers for all endothelial cells (CD31), arteries (α-SMA) and veins/endocardium (EMCN). Blue boxes indicate regions shown in Fig. 3f.
(**b**) Transverse section through an adult Dll4i3mutMEF:*lacZ* transgenic heart, showing this enhancer is not active in the adult heart (representative of at least 5 independent experiments).
Black/white arrows indicate the endocardium, red arrowheads indicate arteries, blue arrowheads indicate veins. rv, right ventricle; lv, left ventricle. Black scale bars represent 500μm.

Supplementary Figure 11

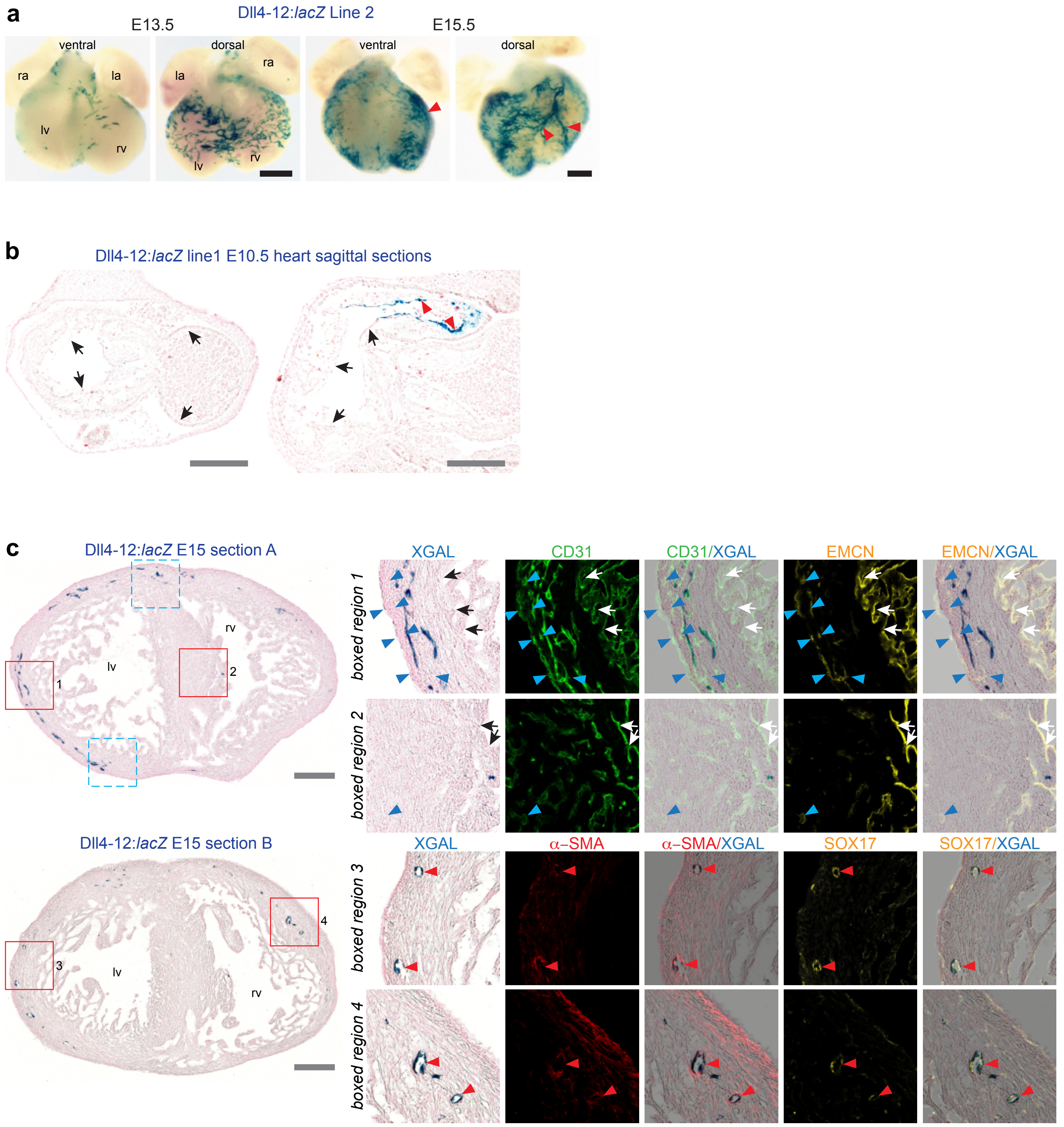

Supplementary Figure 11 relating to Figure 4 in main text.

Further analysis of Dll4-12:*lacZ* enhancer activity in the embryonic heart
(**a**) Representative images (of at least 5 biologically independent samples) of E13.5 and E15.5 hearts from a second, independent line expressing the Dll4-12:*lacZ* transgene. Enhancer activity closely replicates the expression patterns of the hearts shown in Fig. 4b.
(**b**) Representative sagittal sections through the E10.5 Dll4-12:*lacZ* heart (from three biologically independent samples). Activity is seen in outflow tract (red arrowheads) but not endocardium (black arrows).
(**c**) Further views of the E15.5 transverse sections through Dll4-12:*lacZ* hearts shown in Fig. 4c (representative of eight similar biologically independent experiments). Blue X-gal staining shows enhancer activity comparative to markers for all endothelial cells (CD31), arteries (α-SMA and SOX17) and veins/endocardium (EMCN).
Black/white arrows indicate the endocardium, red arrowheads indicate arteries, blue arrowheads indicate veins. Black scale bars represent 500μm, grey scale bars represent 200μm.

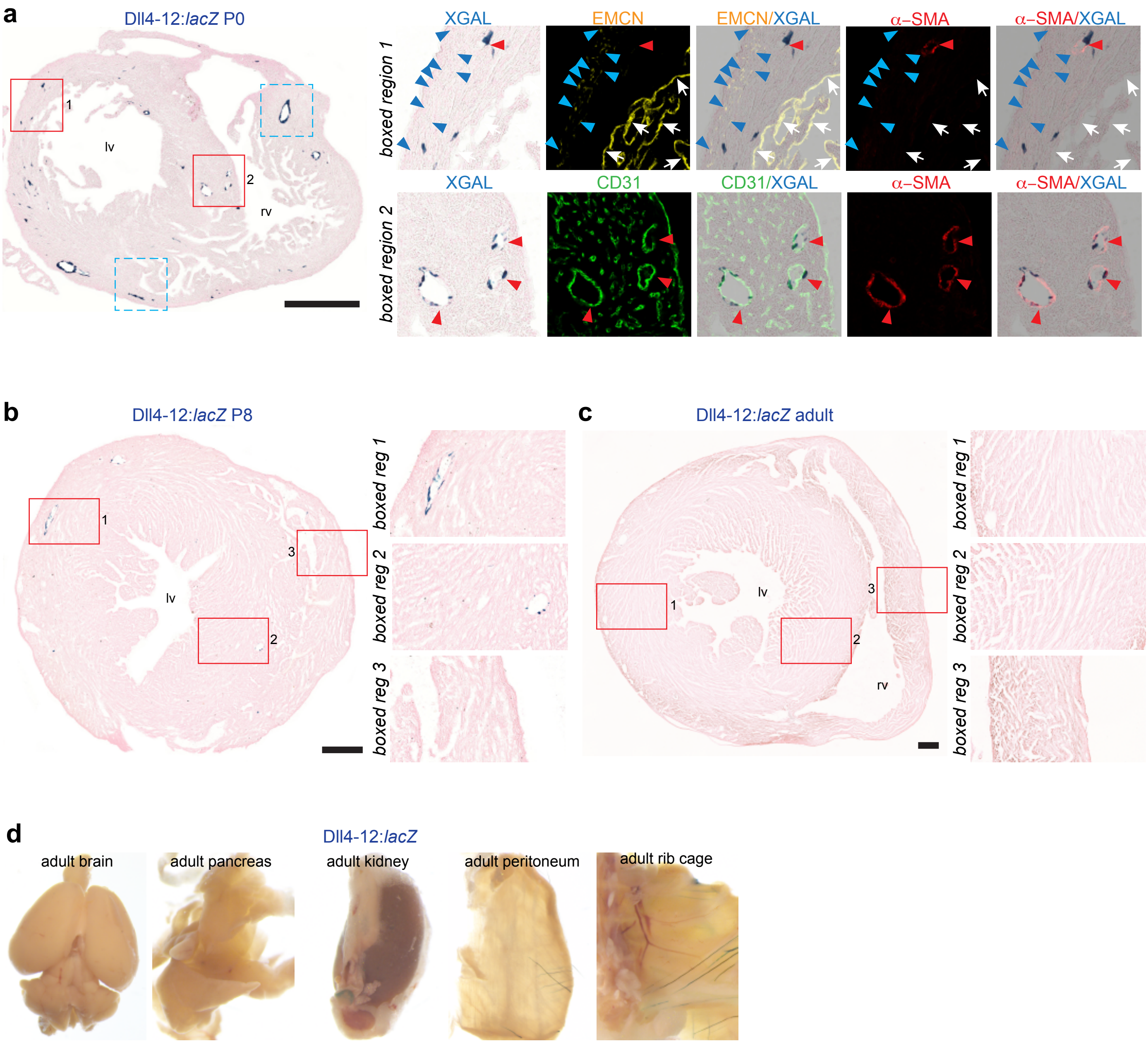
a
Dll4-12:lacZ P0
lv
rv
1
2
XGAL
EMCN
EMCN/XGAL
α–SMA
α–SMA/XGAL
boxed region 1
CD31
CD31/XGAL
boxed region 2
b
Dll4-12:lacZ P8
3
boxed reg 1
boxed reg 2
boxed reg 3
c
Dll4-12:lacZ adult
d
adult brain
adult pancreas
adult kidney
adult peritoneum
adult rib cage

Supplementary Figure 12 relating to Figure 4 in main text

Further analysis of Dll4-12:*lacZ* enhancer activity in the adult
(**a**) Further views of the P0 transverse sections through Dll4-12:*lacZ* hearts shown in Fig. 4d (representative of five similar biologically independent experiments). Blue X-gal staining showing enhancer activity comparative to markers for all endothelial cells (CD31), arteries (α-SMA) and veins/endocardium (EMCN). Blue boxes indicate regions shown in Fig. 4d.
(**b-c**) Representative transverse sections through a P8 (b) and adult (c) Dll4-12:*lacZ* transgenic heart. Dll4-12 enhancer remains active in the P8 heart in the largest coronary arteries, whilst by adulthood the enhancer no longer shows any activity. Representative of at least 5 biologically independent embryos.
(**d**) Dll4-12:*lacZ* activity is not detected in mature arteries in adult organs (organs are from one animal and represent at least 3 biologically independent animals) .
Black/white arrows indicate the endocardium, red arrowheads indicate arteries, blue arrowheads indicate veins rv, right ventricle; lv, left ventricle. Black scale bars represent 500μm.

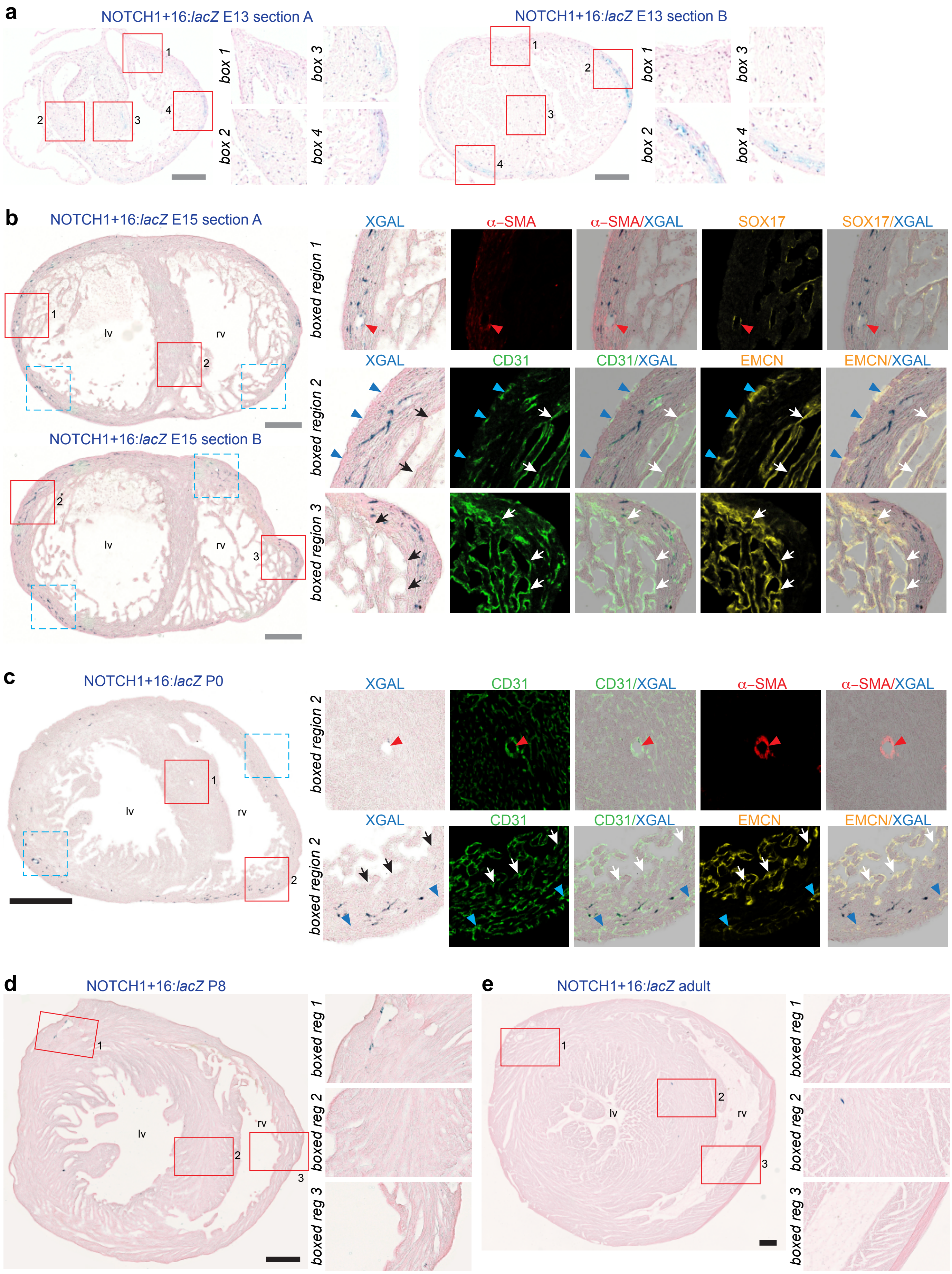
a
NOTCH1+16:lacZ E13 section A
box 1
box 2
box 3
box 4
NOTCH1+16:lacZ E13 section B
b
NOTCH1+16:lacZ E15 section A
NOTCH1+16:lacZ E15 section B
lv
rv
XGAL
α–SMA
α–SMA/XGAL
SOX17
SOX17/XGAL
CD31
CD31/XGAL
EMCN
EMCN/XGAL
boxed region 1
boxed region 2
boxed region 3
c
NOTCH1+16:lacZ P0
d
NOTCH1+16:lacZ P8
boxed reg 1
boxed reg 2
boxed reg 3
e
NOTCH1+16:lacZ adult

Supplementary Figure 13 relating to Figure 5 in main text

Further analysis of NOTCH1+16:*lacZ* enhancer activity in the embryonic and postnatal heart

(**a**) Representative transverse sections through E13.5 NOTCH1+16:*lacZ* transgenic hearts (representative of at least 5 biologically independent embryos). No activity is seen in the septum regions.

(**b-c**) Further views of the transverse sections through the E15.5 (b) and P0 (c) NOTCH1+16:*lacZ* transgenic hearts shown in Fig. 5c-d (representative of five (b) and four (c) similar biologically independent experiments). Blue X-gal staining shows enhancer activity compared with markers for all endothelial cells (CD31), arteries (SOX17 and α-SMA) and veins/endocardium (EMCN). Blue boxes indicate regions shown in Fig. 5.

(**d-e**) Representative transverse sections through a P8 (d) and adult (e) NOTCH1+16:*lacZ* transgenic heart (representative of at least 5 biologically independent animals). Some arterial X-gal staining is seen at P8 but lost in the adult heart. Black/white arrows indicate the endocardium, red arrowheads indicate arteries, blue arrowheads indicate veins. rv, right ventricle; lv, left ventricle. Black scale bars represent 500μm, grey scale bars represent 200μm.

Supplementary Figure 14

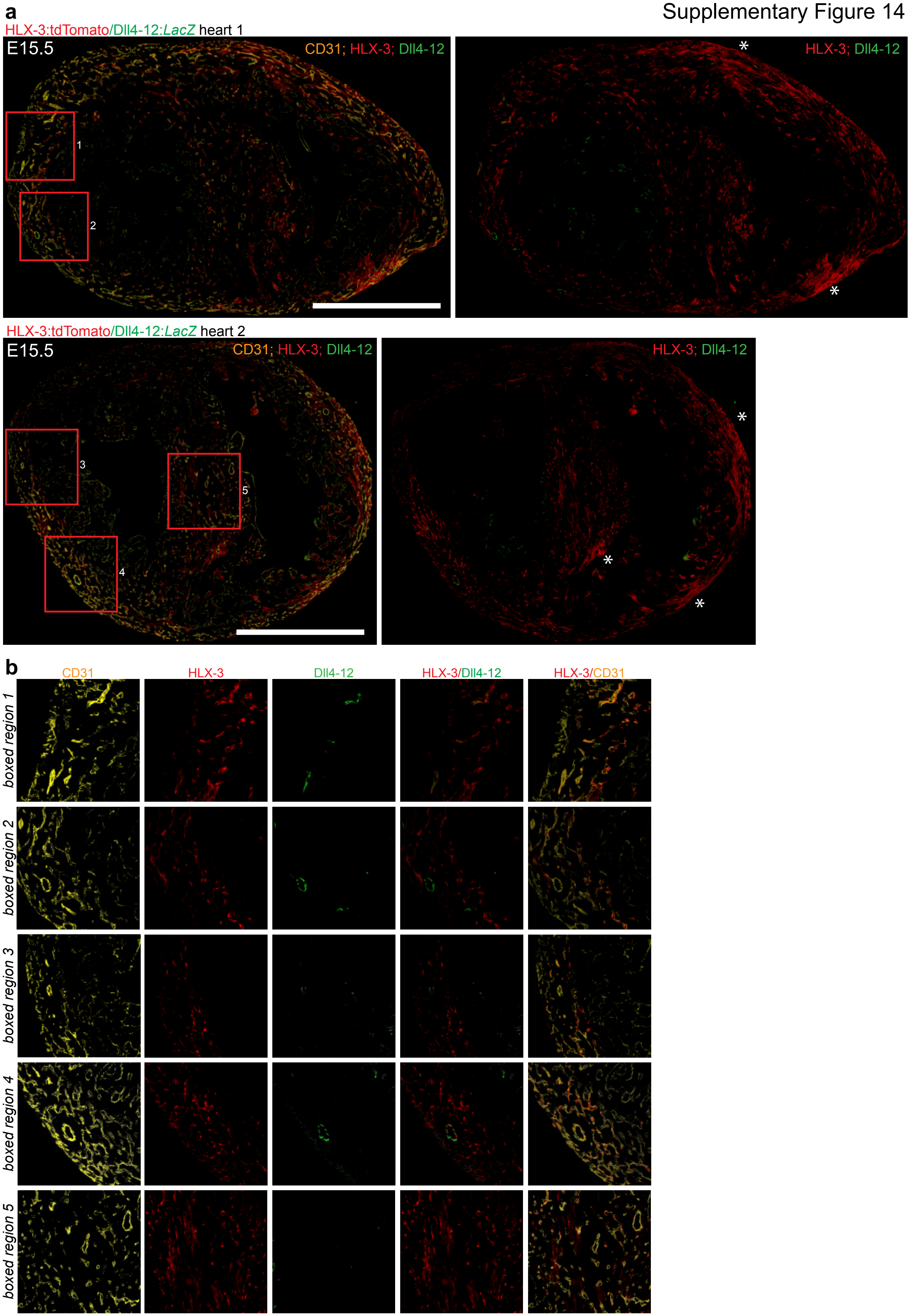

Supplementary Figure 14 relating to Figure 6 in main text

Further images of hearts expressing both HLX-3:tdTomato and Dll4-12:*lacZ* transgenes at E15.5

Images of entire cross-section images of two different HLX-3:tdTomato;Dll4-12:*lacZ* E15.5 hearts (**a**) and focused regions within these sections (**b**). The same sections are shown in Fig. 6 at higher magnification. Immunostaining for CD31, ß-gal and tdTomato demonstrate patterns of transgene activity comparative to all endothelial cells. Ectopic activity of tdTomato was seen in some myocardium (*). White scale bars represent 500μm.

Supplementary Figure 15

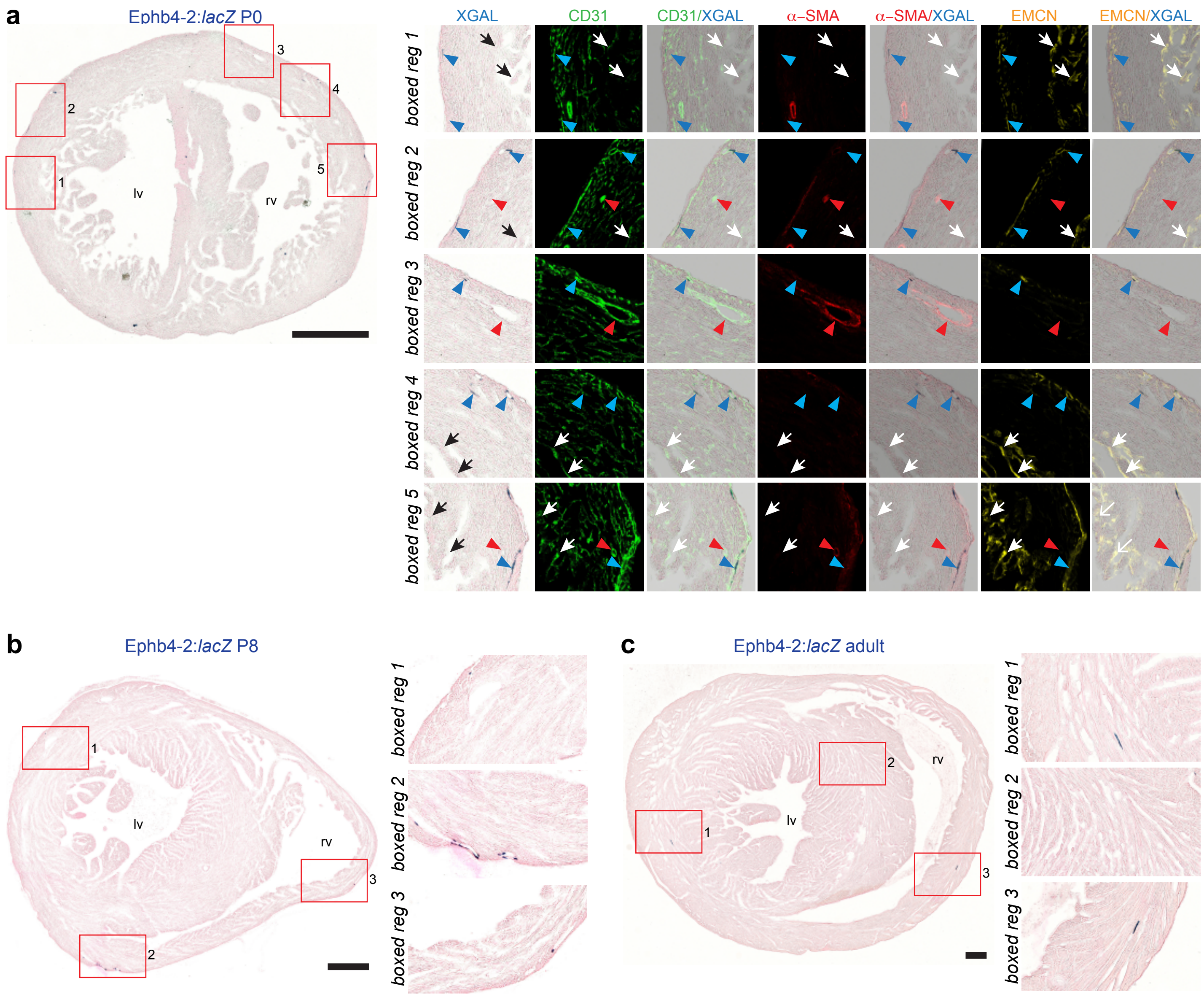

Supplementary Figure 15 relating to Figure 7 in main text

Further analysis of EphB4-2:*lacZ* enhancer activity in the postnatal heart

(**a**) Transverse section through the entire P0 EphB4:*lacZ* transgenic heart shown in Figure 7d (representative of three similar biologically independent experiments). Enhancer activity represented by blue X-gal staining compared to markers for endothelium (CD31), arteries (α-SMA) and veins/endocardium (EMCN).

(**b-c**) Transverse sections through a P8 (b) and adult (c) EphB4-2:*lacZ* transgenic heart, representative of at least 5 biologically independent animals. Some EphB4-2 enhancer activity remains in the larger veins in the P8 heart, whilst very little enhancer activity is seen in the adult heart.

Black/white arrows indicate the endocardium, red arrowheads indicate arteries, blue arrowheads indicate veins. rv, right ventricle; lv, left ventricle. Black scale bars represent 500μm.

Supplementary Figure 16

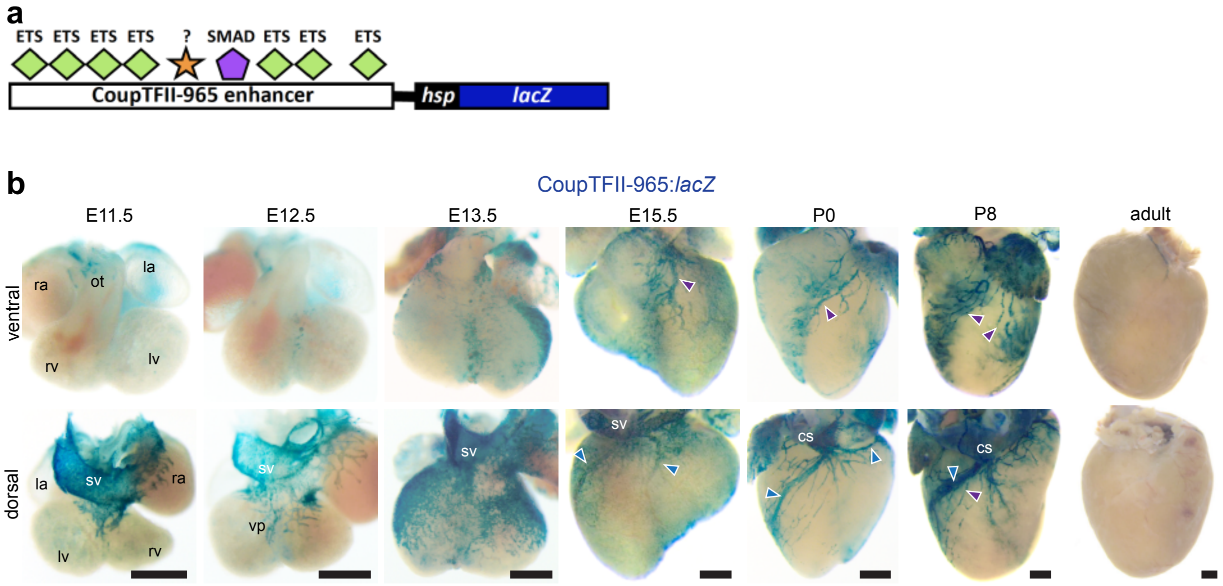

Supplementary Figure 16 relating to Figure 7 in main text

The CoupTFII-965 SMAD-driven enhancer drives reporter gene expression in the SV-derived vascular plexus, coronary veins and coronary lymphatics

(**a**) Schematic showing the CoupTFII-965 enhancer:*lacZ* transgene, with verified binding motifs for ETS and SMAD transcription factor represented by coloured shapes[1]. The star shape represents the unknown transcription factor(s) required for lymphatic endothelial expression of CoupTFII-965.

(**b**) Whole-mount images of CoupTFII-965:*lacZ* transgenic hearts from E11.5 through to adulthood, with enhancer activity detected by blue X-gal staining (each representative of at least 5 biologically representative samples. The CoupTFII-965:lacZ transgene is active in the SV and the early SV-derived vascular plexus as it migrates down the dorsal aspect of the embryonic heart at E11.5-E13.5. From E15.5, transgene activity becomes restricted to the developing coronary veins and the coronary lymphatics, which is still clearly seen in the postnatal P8 heart. However, little transgene activity was detected in the adult heart. rv, right ventricle; lv, left ventricle; ra, right atrium; la, left atrium; ot, outflow tract; sv, sinus venosus; vp, vascular plexus. Blue arrowheads indicate veins, purple arrowheads indicate lymphatic vessels. Black scale bars represent 500μm.

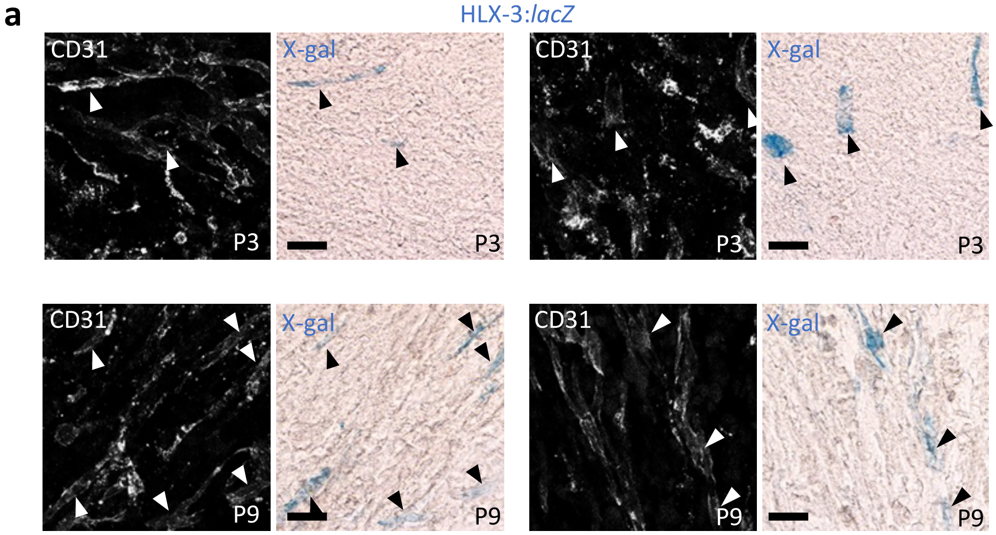
a
HLX-3:lacZ
CD31
X-gal
P3
P9

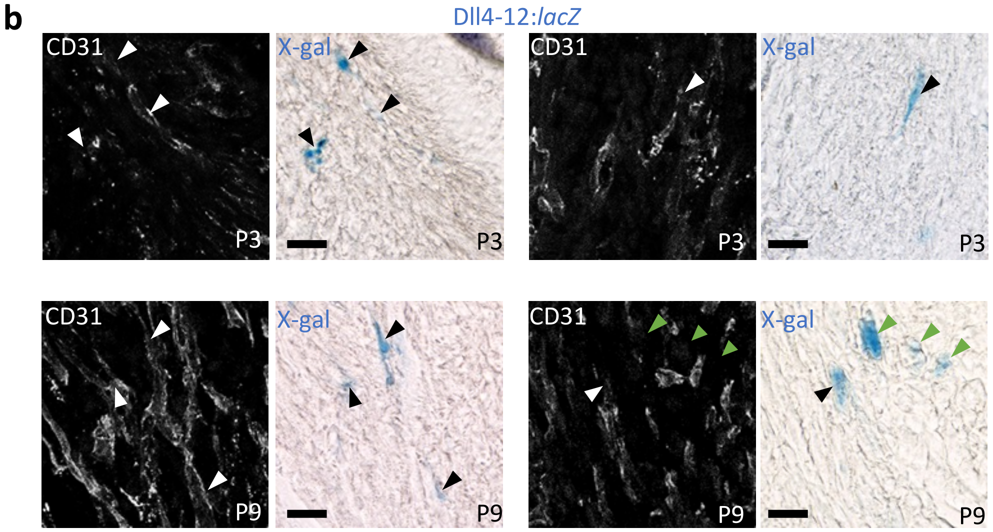
b
Dll4-12:lacZ
CD31
X-gal
P3
P9

Supplementary Figure 17 relating to Figures 8 and 9 in main text

Enhancer:*lacZ* transgene expression correlates with endothelial markers in post-MI neonatal hearts

Representative slides stained for X-gal comparative to the pan-endothelial marker CD31 demonstrate HLX-3:*lacZ* (**a**) and Dll4-12:*lacZ* (**b**) expression in endothelial cells in the hearts two days after MI (representative of three similar biologically independent samples). Black/white arrowheads indicate cells expressing β-gal, green arrowheads indicate ectopic expression in non-CD31 expressing cells, black scale bars represent 20μm.

Supplementary Figure 18

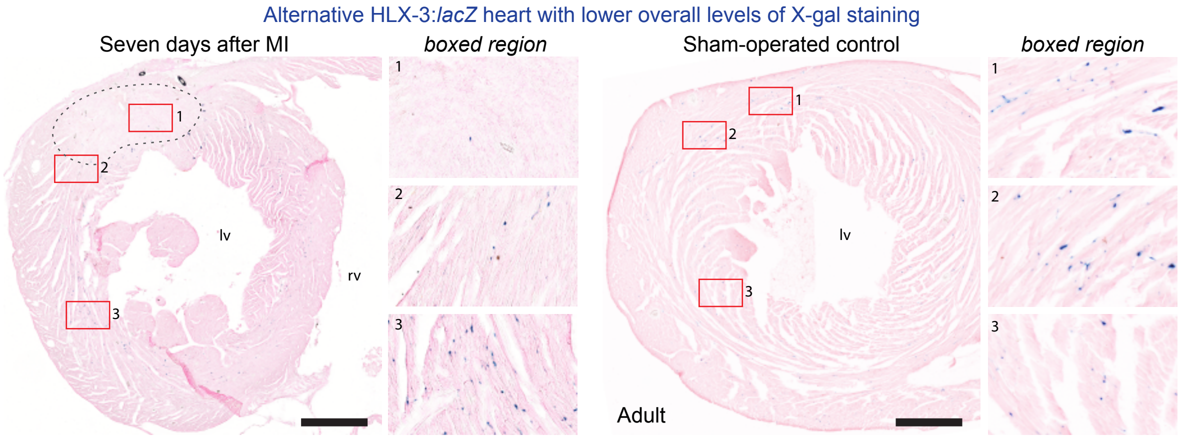

Supplementary Figure 18 relating to Figure 10 in main text

Adult HLX-3:*lacZ* transgenic hearts with less robust transgene expression show the same pattern of transgene repression after MI

MI was conducted as in Fig. 10, infarct regions are indicated by grey dashed line. Magnified views are shown of regions within the injury (box 1), at the injury border zone (box 2) and remote from the injury (box 3), and can be compared to sham-operated day seven controls. lv, left ventricle; rv, right ventricle. Black scale bars represent 500µm.

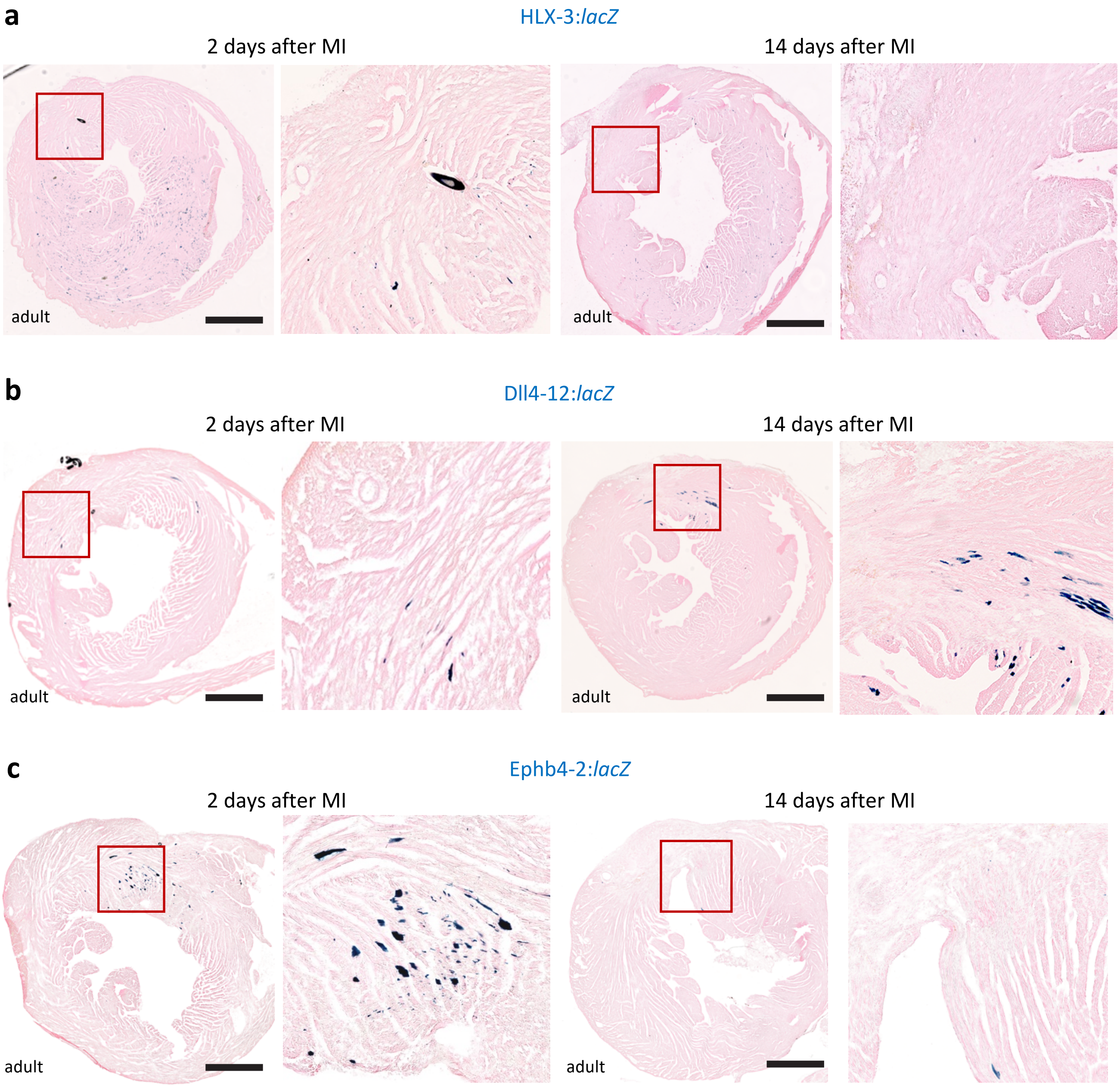
a
HLX-3:*lacZ*
2 days after MI
14 days after MI
adult
adult
b
Dll4-12:*lacZ*
2 days after MI
14 days after MI
adult
adult
c
Ephb4-2:*lacZ*
2 days after MI
14 days after MI
adult
adult

Supplementary Figure 19 relating to Figure 10 in main text

Analysis of HLX-3:*lacZ,* Dll4-12:*lacZ* and Ephb4-2:*lacZ* transgene activity in adult hearts two and 14 days post-MI

Transgenic HLX-3:*lacZ* (**a**), Dll4-12:*lacZ* (**b**) and Ephb4:*lacZ* (**c**) hearts two and 14 days after MI surgery in the adult heart. For all enhancers, expression patterns were similar to those seen seven days after MI in Fig. 5. N=4 biologically independent experiments for each timepoint for each transgene. Black scale bars represent 500μm.

Supplementary Figure 20

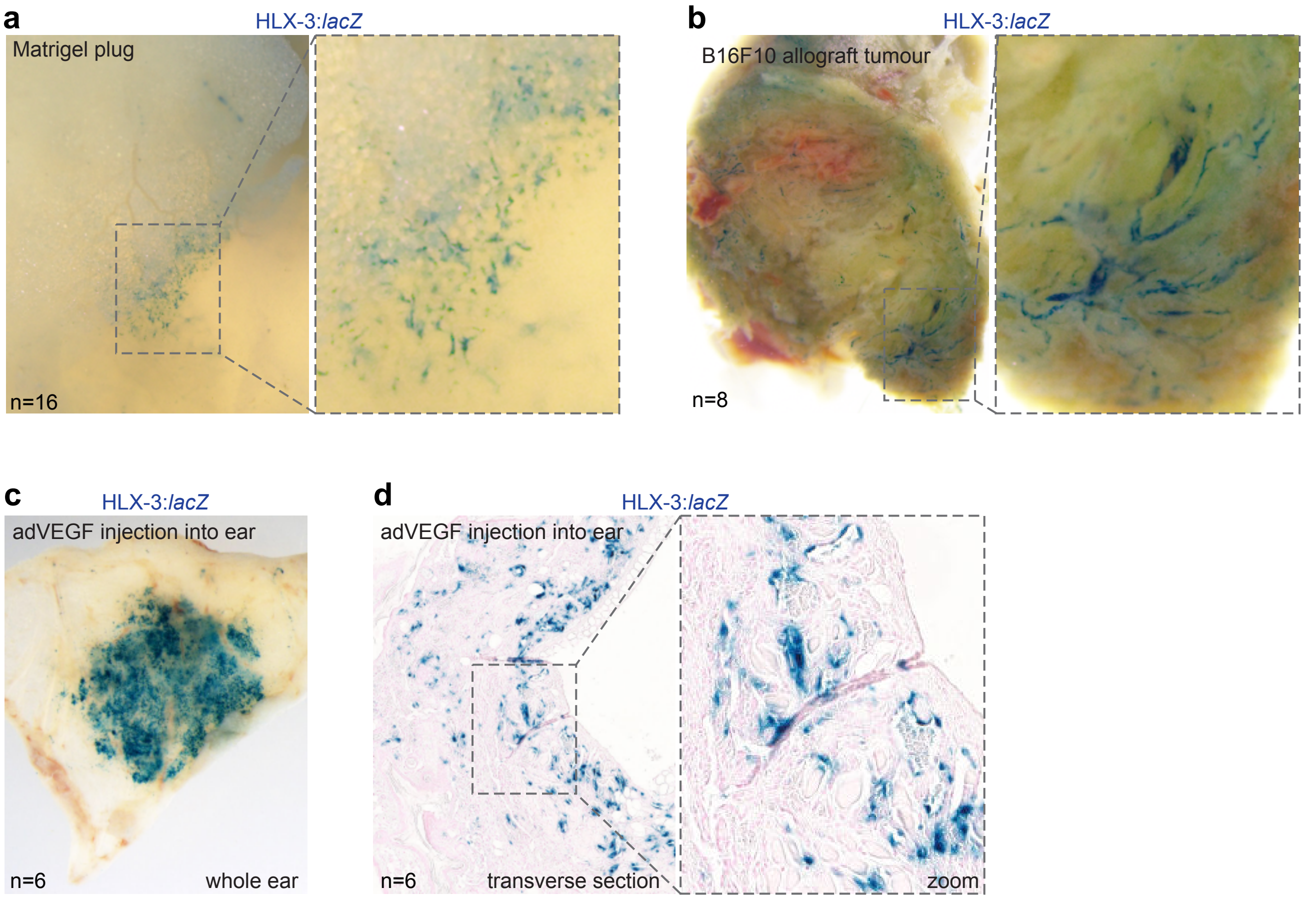

Supplementary Figure 20 relating to Figure 10 in main text

Analysis of HLX-3:*lacZ* transgene in other models of adult angiogenesis

Representative images of HLX-3:*lacZ* transgene expression during neovascularization in a Matrigel plug (**a**), B16F10 melanoma tumour (**b**) and ten days after adVEGFA^{165} injection into a Foxn1$^{-/-}$;HLX-3:*lacZ* mouse ear (**c**, whole ear and **d**, transverse sections through the ear). In all circumstances, the HLX-3:*lacZ* transgene is reactivated. N numbers of biologically independent samples indicated on image.

Supplementary Figure 21

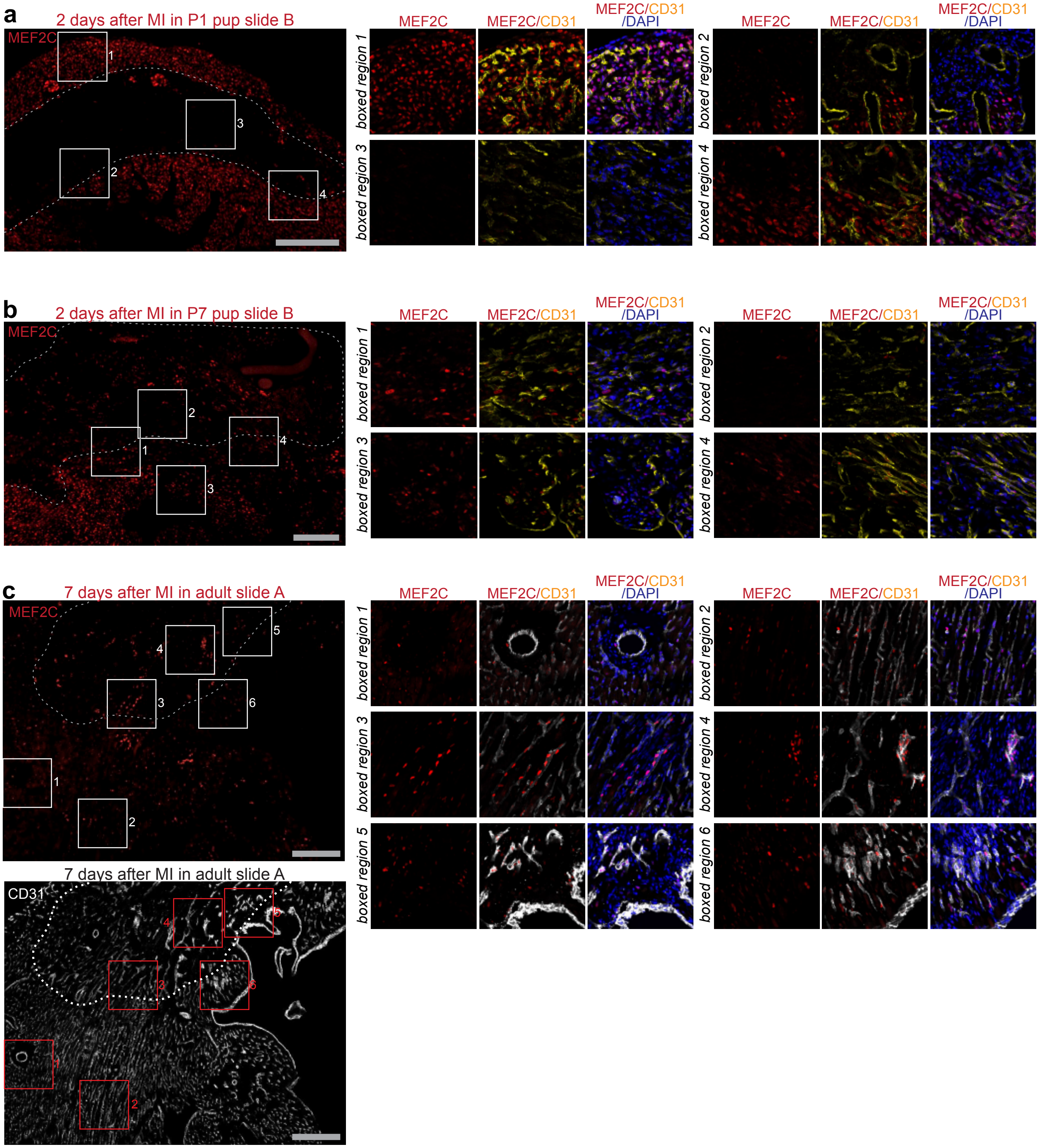

Supplementary Figure 21 relating to Figures 8-10 in main text

MEF2C expression profile in P3, P9 and adult hearts after MI
(**a-b**) Two days after MI in a P1 (a) and P7 (b) pup, MEF2C expression is found widely in the heart, although excluded from the infarct region. Away from the infarct, MEF2C is seen in the majority of CD31-positive nuclei. Both images representative of three similar biological independent experiments.
(**c**) Seven days after MI in adult hearts, MEF2C is found in a subset of CD31-positive endothelium, including in regions close to infarct. Dashed white lines indicate the injured myocardium. Grey scale bars represent 200μm. Images represent five of nine biologically independent experiments, less intense MEF2C staining was seen in remaining four experiments.

Supplementary Figure 22

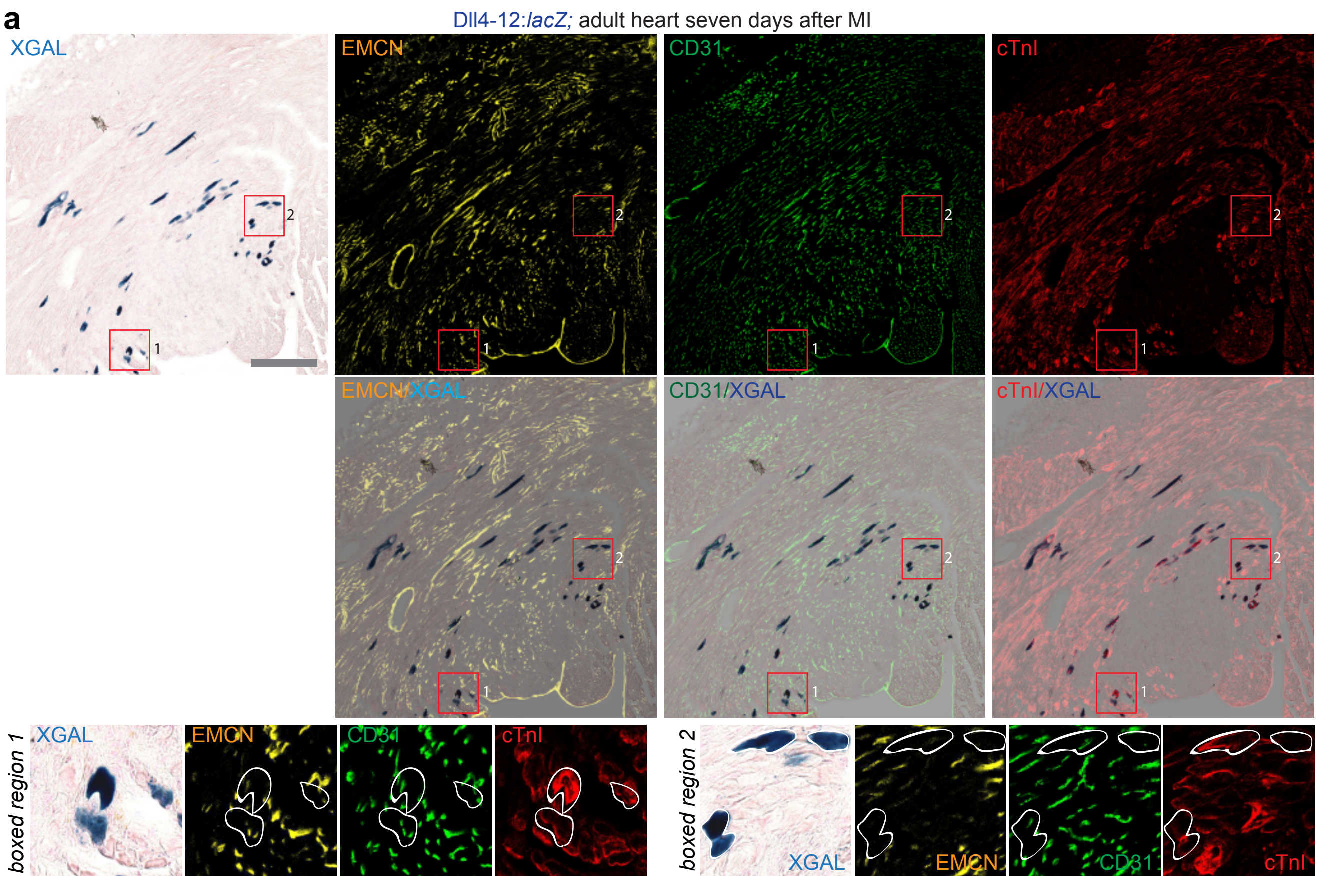

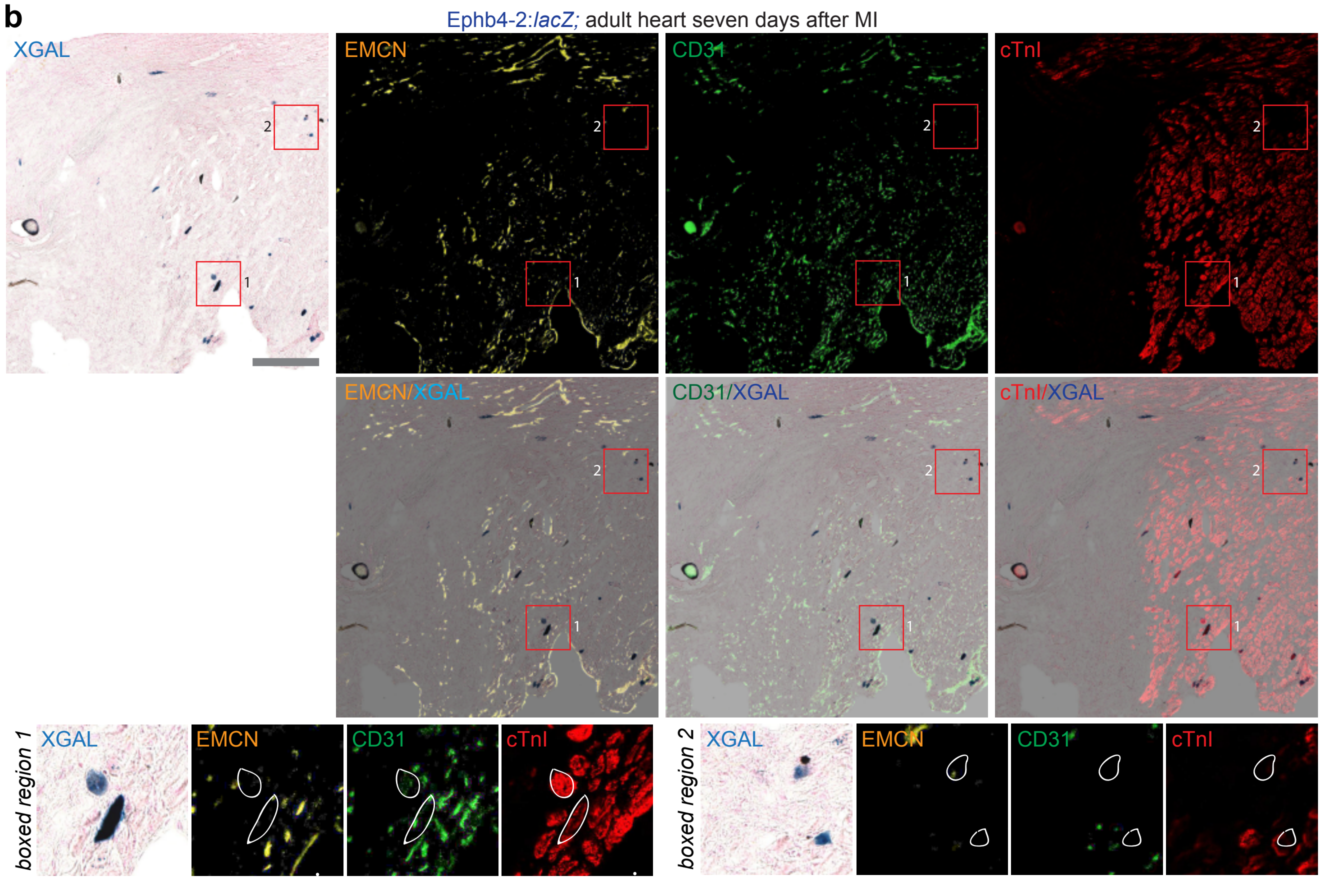

Supplementary Figure 22 relating to Figure 10 in main text

Expression of the Dll4-12:*lacZ* and Ephb4-2:*lacZ* transgenes in the adult heart post-MI does not correlate with vascular markers

The expression of Dll4-12:*lacZ* (**a**) and Ephb4-2:*lacZ* (**b**) in post-MI adult hearts is shown as X-gal staining comparative to vascular markers EMCN and CD31 and cardiomyocyte marker cTnI around the infarct region, each image representative of five (a) and three (c) similar biologically independent experiments). Little X-gal staining co-expresses with endothelial markers. Grey scale bars represent 200μm.

Supplementary Figure 23

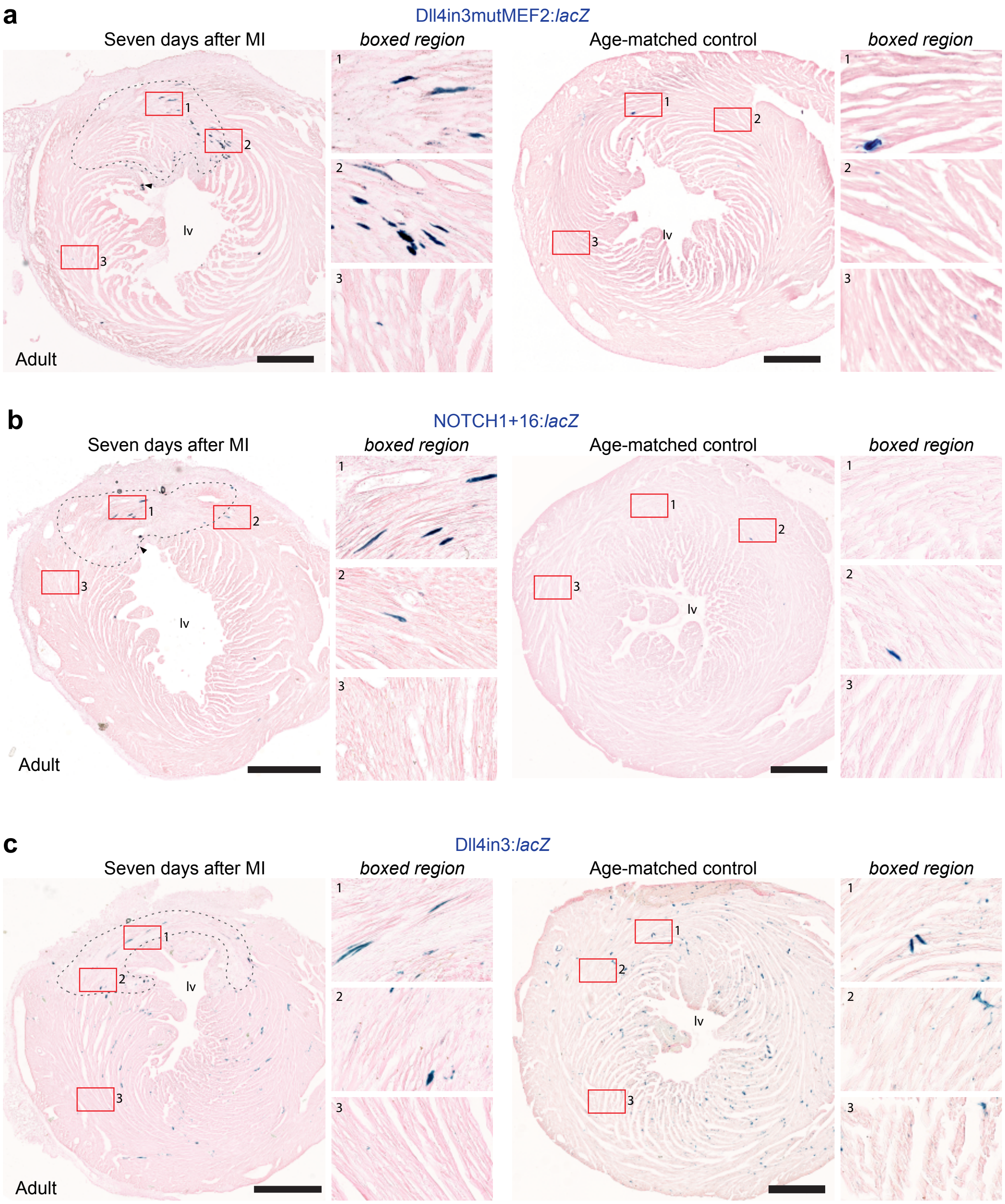

Supplementary Figure 23 relating to Figure 10 in main text

Analysis of other vascular enhancers in the post-MI adult heart
Permanent ligation of the left anterior descending coronary artery was performed as in Figure 10. Representative sections through Dll4in3mutMEF2:*lacZ* (**a**, n=4 biologically independent animals), NOTCH1+16:*lacZ* (**b,** n=4 biologically independent animals), and Dll4in3:*lacZ* (**c**, n=5 biologically independent animals) hearts collected seven days after MI surgery compared to sham-operated controls. Infarct regions are indicated by grey dashed line, transgene expression is detected by X-gal staining (blue). Magnified views are shown of regions within the injury (box 1), at the injury border zone (box 2) and remote from the injury (box 3), and can be compared to sham-operated day seven controls. lv, left ventricle; rv, right ventricle. Black scale bars represent 500μm.

<u>Supplementary Reference</u>

1. Neal, A. *et al.* Venous identity requires BMP signalling through ALK3. *Nat Commun* **10,** 453 (2019).
