# Supplementary material for: Regulatory pathways governing murine coronary vessel formation are dysregulated in the injured adult heart
Source: Nat Commun. 2019 Jul 22;10:3276. doi: 10.1038/s41467-019-10710-2 (PMC6646353; doi:10.1038/s41467-019-10710-2)
Supplement: Supplementary file 2 — Reporting Summary [file 41467_2019_10710_MOESM2_ESM.pdf]

natureresearch

Corresponding author(s): Sarah De Val

Reporting Summary

Nature Research wishes to improve the reproducibility of the work that we publish. This form provides structure for consistency and transparency in reporting. For further information on Nature Research policies, see Authors & Referees and the Editorial Policy Checklist.

Statistical parameters

When statistical analyses are reported, confirm that the following items are present in the relevant location (e.g. figure legend, table legend, main text, or Methods section).

n/a	Confirmed	
☐	☒	The exact sample size (*n*) for each experimental group/condition, given as a discrete number and unit of measurement
☐	☒	An indication of whether measurements were taken from distinct samples or whether the same sample was measured repeatedly
☒	☐	The statistical test(s) used AND whether they are one- or two-sided<br>*Only common tests should be described solely by name; describe more complex techniques in the Methods section.*
☒	☐	A description of all covariates tested
☒	☐	A description of any assumptions or corrections, such as tests of normality and adjustment for multiple comparisons
☒	☐	A full description of the statistics including central tendency (e.g. means) or other basic estimates (e.g. regression coefficient) AND variation (e.g. standard deviation) or associated estimates of uncertainty (e.g. confidence intervals)
☒	☐	For null hypothesis testing, the test statistic (e.g. *F*, *t*, *r*) with confidence intervals, effect sizes, degrees of freedom and *P* value noted<br>*Give P values as exact values whenever suitable.*
☒	☐	For Bayesian analysis, information on the choice of priors and Markov chain Monte Carlo settings
☒	☐	For hierarchical and complex designs, identification of the appropriate level for tests and full reporting of outcomes
☒	☐	Estimates of effect sizes (e.g. Cohen's *d*, Pearson's *r*), indicating how they were calculated
☒	☐	Clearly defined error bars<br>*State explicitly what error bars represent (e.g. SD, SE, CI)*

Our web collection on statistics for biologists may be useful.

Software and code

Policy information about availability of computer code

Data collection	Microscope images were analysed with ZEN, ImageJ and Adobe photoshop.
Data analysis	

For manuscripts utilizing custom algorithms or software that are central to the research but not yet described in published literature, software must be made available to editors/reviewers upon request. We strongly encourage code deposition in a community repository (e.g. GitHub). See the Nature Research guidelines for submitting code & software for further information.

Data

Policy information about availability of data

All manuscripts must include a data availability statement. This statement should provide the following information, where applicable:

- Accession codes, unique identifiers, or web links for publicly available datasets
- A list of figures that have associated raw data
- A description of any restrictions on data availability

The authors declare that the main data supporting the findings of this study are available within the article, its Supplementary Figures and Methods. Extra data are available from the corresponding author upon request.

Field-specific reporting

Please select the best fit for your research. If you are not sure, read the appropriate sections before making your selection.

☒ Life sciences ☐ Behavioural & social sciences ☐ Ecological, evolutionary & environmental sciences

For a reference copy of the document with all sections, see nature.com/authors/policies/ReportingSummary-flat.pdf

Life sciences study design

All studies must disclose on these points even when the disclosure is negative.

Sample size	Mouse: Only transgenic mice lines already created and described in previous publications were used. Transgenic mice used were all from established lines previously described. Therefore, no variation was expected nor seen in transgene expression patterns. The exception was HLX-3:lacZ activity in adult mice, which showed intra- and inter-litter variation in intensity but no variation in expression pattern. This is represented in the relevant figures by representative pictures of each outcome. No experimental randomization or blinding was used as this was not considered necessary. For all MI analysis, the minimum number of repeats at a single analysis time-point was 4 different animals. For adult MI analysis, each transgene was investigated in at least 12 separate animals after surgery, with at least 4 animals investigated at each time-point.
Data exclusions	In post-natal analysis of hearts, additional tissue was taken and X-gal stained to confirm genotype. In the rare event that lacZ-positive animals had no detectable staining in tissues away from the heart after MI, errors in genotyping were assumed and the sample was excluded from analysis.
Replication	All experimental findings were reliably reproduced at least 3 times unless otherwise clearly stated in the manuscript
Randomization	There was no randomization used
Blinding	No experimental blinding was used as phenotypes of control and treated embryos were easily detectable.

Reporting for specific materials, systems and methods

Materials & experimental systems

n/a	Involved in the study	
☒	☐	Unique biological materials
☐	☒	Antibodies
☒	☐	Eukaryotic cell lines
☒	☐	Palaeontology
☐	☒	Animals and other organisms
☒	☐	Human research participants

Methods

n/a	Involved in the study	
☒	☐	ChIP-seq
☒	☐	Flow cytometry
☒	☐	MRI-based neuroimaging

Antibodies

Antibodies used	All antibodies used are commercially available. Primary antibodies used were: rat anti-CD31 (1:200, Dianova DIA-310), armenian hamster anti-CD31 (1:100, Abcam ab119341), biotinylated Isolectin B4 (1:200, Vector Laboratories B-1205), rat anti-Endomucin (1:50, Santa Cruz sc-65495), goat anti-DLL4 (1:50, R&D Systems AF1389), goat anti-EphB4 (1:50, R&D Systems AF446), mouse anti-Actin, α-Smooth Muscle - Cy3™ (1:200, Sigma-Aldrich C6198), mouse anti-Actin, α-Smooth Muscle (1:300, Sigma-Aldrich A5228), goat anti-SOX17 (1:300, R&D Systems AF1924), rabbit anti-Cardiac Troponin I (1:100, Abcam ab47003), rabbit anti-RFP (1:1000, Rockland 600-401-379, used to detect tdTomato), chicken anti-β-galactosidase (1:500, Abcam ab9361), rabbit anti-MEF2A (1:100, Abcam ab109420), rabbit anti-MEF2C (1:1000, Cell Signalling 5030S) and rabbit anti-MEF2D (1:1000, Abcam ab32845).
Validation	All antibodies used are commercially available and are validated for the species and technique for which they were used. Validation data is easily accessible for all antibodies on the website of the manufacturing company by searching the product numbers that are all provided above.

Animals and other organisms

Policy information about studies involving animals; ARRIVE guidelines recommended for reporting animal research

Laboratory animals	Mouse, mus musculus: embryonic day 10.5 to adult were used in analysis, sex unknown, and are a mixed strain of C57BL6 and CBA (each transgenic mouse line is originally made on a c57Bl/6J crossed with CBA/J background, but subsequent generations are back-crossed with pure C57BL/6J mice) .
Wild animals	N/A
Field-collected samples	N/A
